# From mind to match: the impact of psychological skills intervention programs on football players’ mental toughness, decision-making, and tactical skills: a randomized controlled group study

**DOI:** 10.3389/fpsyg.2026.1828902

**Published:** 2026-04-10

**Authors:** İbrahim Dalbudak, Mehmet Behzat Turan, Oğulcan Usuflu, Barış Karaoğlu, Osman Pepe, Melih Balyan, Mustafa Kara, Seda Evyapan Aydin

**Affiliations:** 1 Faculty of Sports Sciences, Uşak University, Uşak, Türkiye; 2 Faculty of Sports Sciences, Erciyes University, Kayseri, Türkiye; 3 Department of Management and Organization, Vocational School, Istanbul Rumeli University, Istanbul, Türkiye; 4 Faculty of Sports Sciences, Bingol University, Bingöl, Türkiye; 5 Faculty of Sports Sciences, Süleyman Demirel University, Isparta, Türkiye; 6 Faculty of Sports Sciences, Ege University, İzmir, Türkiye; 7 Institute of Health Sciences, Celal Bayar University, Manisa, Türkiye; 8 Institute of Graduate Studies, Uşak University, Uşak, Türkiye

**Keywords:** decision-making, football, mental toughness, players, psychological skills training, tactical skills

## Abstract

**Background:**

Football is characterized by rapidly changing and highly demanding competitive conditions in which players are frequently exposed to substantial psychological pressure. Athletes’ psychological responses to these pressures can directly influence critical performance-related factors such as decision-making, tactical execution, and overall game performance. In modern football, physical and technical abilities alone are often insufficient for achieving high-level performance; psychological competencies have become increasingly important determinants of success. Consequently, the development and implementation of structured psychological training approaches have gained considerable attention in sports science research. In this context, Psychological Skills Intervention (PSI) programs are considered promising strategies for enhancing athletes’ psychological capacities and optimizing their performance in competitive environments.

**Objective:**

This study aims to investigate the effects of Psychological Skills Intervention (PSI) programs on football players’ mental toughness, decision-making abilities, and tactical skills.

**Methods:**

The study sample consisted of football players from the youth academies of professional football clubs competing in the Turkish Football Federation (TFF) Development Leagues during the 2025–2026 season. A total of 55 football players voluntarily participated in the study and were divided into a control group (*n* = 28) and an experimental group (*n* = 27). The control group followed the standard training program implemented by their teams. In contrast, the experimental group participated in an eight-week Psychological Skills Intervention (PSI) program in addition to the standard training program. The variables included in the study were assessed at three different time points. The data from the study were analyzed using SPSS 26. Repeated Measures ANOVA with Bonferroni *post hoc* comparisons was conducted to examine within-group changes over time, while Independent Samples t-tests were used to evaluate differences between the control and experimental groups.

**Results:**

Although improvements were observed in the mental toughness, decision-making, and tactical skills of U-19 footballers in the control group, statistically significant improvements were observed in the experimental group who participated in the PSI program.

**Conclusion:**

The findings suggest that integrating Psychological Skills Intervention (PSI) programs as a systematic, integral component of training for footballers competing in development leagues can significantly enhance players’ individual performance and career development as they progress through the competitive pathway. Furthermore, such programs may play a critical role in developing elite-level footballers capable of contributing to the long-term and sustainable success of national football.

## 1 Introduction

Football is one of the most popular sports worldwide and continues to attract millions of participants and spectators across different cultures and societies (Ergen, 2018; Worsey et al., 2020). Over time, football has evolved from a purely athletic activity into a multidimensional global industry characterized by complex economic, social, and cultural dynamics. This transformation has been driven by multiple factors, including sponsorship agreements, broadcasting rights, media influence, and the growing engagement of global fan communities (Pepe et al., 2025). Within this expanding ecosystem, the on-field performance of football players remains the central determinant of competitive success and plays a critical role in maintaining the sustainability and long-term development of the sports industry.

Traditionally, success in football was primarily associated with physical fitness and technical proficiency (Gülşen, 2008; Uğraş et al., 2002). However, contemporary sport science literature increasingly emphasizes that athletic performance is also strongly influenced by psychological competencies and mental skills (Wittig, 1984; Ansoy and Pepe, 2021; Stojmenović and Stojmenović, 2023; Kalinowski et al., 2020; Arısoy et al., 2020; Kurt and Yaman, 2025). The modern game has become faster, more tactically demanding, and increasingly unpredictable, requiring athletes to process complex information rapidly and make accurate decisions under pressure. In this context, psychological attributes such as attentional control, emotional regulation, confidence, and cognitive flexibility have emerged as essential components of elite football performance (Weinberg and Gould, 2019).

The development of successful football players is therefore widely recognized as a long-term and multidimensional process that involves the systematic integration of physical, technical, tactical, and psychological elements (Ermiş, 2019). This holistic perspective aligns with contemporary theories of athlete development, which highlight the importance of cognitive and psychological processes in supporting expertise and performance consistency (Ericsson et al., 1993). In team sports such as football, athletes must continuously perceive environmental information, interpret game situations, and adjust their actions accordingly. According to the ecological dynamics framework, decision-making in sport emerges from the dynamic interaction between the athlete and the surrounding performance environment (Araújo et al., 2006). This perspective suggests that perceptual-cognitive skills and psychological readiness are fundamental in facilitating effective perception–action coupling during gameplay.

Psychological skills such as imagery, attentional focus, emotional control, and self-talk can enhance athletes’ ability to process environmental cues and respond effectively to changing game situations. These skills help players maintain concentration, regulate stress, and execute tactical decisions under high-pressure conditions (Weinberg and Gould, 2019; Zimmerman, 2000). In dynamic and time-constrained team sports environments, the ability to sustain cognitive control and psychological stability often differentiates successful players from less effective performers.

Within the football development pathway, the U19 category (18–19 years old) represents a particularly critical transitional stage. At this age, athletes typically approach the completion of their physical development, while their technical abilities become more stable and their tactical understanding begins to integrate more closely with cognitive decision-making processes (Türkiye Futbol Federasyonu, 2024). As a result, this developmental phase is considered highly important for strengthening psychological resilience, improving cognitive processing speed, and refining decision-making abilities in competitive environments (Benítez-Sillero et al., 2021). Moreover, athletes at this stage often face increased competitive demands and the psychological pressures associated with the transition from youth to professional football. Consequently, mental toughness, effective decision-making, and tactical awareness become critical determinants of performance and long-term career progression (Pires et al., 2025).

In response to these demands, psychological skills intervention (PSI) programs have gained increasing attention in sport psychology research and applied practice. These programs are structured interventions that incorporate techniques such as goal setting, imagery (visualization), self-talk, attentional control, and stress management strategies. Previous studies have demonstrated that such interventions can enhance athletes’ mental toughness, improve their ability to make decisions under pressure, and strengthen tactical awareness during competition (Yılmaz and Abakay, 2025; Gül Filik and Uz Baş, 2024; Kesler et al., 2026; Karademir et al., 2026). By systematically developing psychological competencies, these interventions contribute to the improvement of both individual performance and overall team effectiveness.

Psychological skills training (PST) represents a systematic and evidence-based approach designed to enhance athletes’ mental capacities, including goal setting, imagery, emotional regulation, and attentional control (Weinberg and Gould, 2019). These psychological abilities are closely associated with self-regulation processes that enable athletes to manage stress, maintain concentration, and execute appropriate tactical responses during competition (Zimmerman, 2000). In highly dynamic team sports such as football, where decision-making opportunities emerge rapidly and under pressure, psychological preparedness becomes a key factor shaping athletes’ behavioral responses and tactical effectiveness (Araújo et al., 2019a,b).

Tactical performance in football refers to players’ ability to select and implement appropriate actions according to the demands of the game context, including positioning, passing decisions, spatial awareness, and coordinated team movements (Memmert, 2015). Tactical expertise is influenced not only by physical and technical capabilities but also by perceptual-cognitive and psychological processes that guide athletes’ interpretation of game situations and their subsequent decision-making behavior. Consequently, understanding the role of psychological skills in shaping tactical performance represents an important research area in contemporary sport science.

### 1.1 Mental toughness

When individuals encounter adverse or challenging situations, they initially tend to experience negative emotional responses such as stress, fear, or anxiety. Nevertheless, many individuals are capable of adapting to stressful events and circumstances that may significantly influence their lives. One of the key psychological mechanisms facilitating this adaptation is mental toughness. Mental toughness is described as a dynamic and ongoing developmental process that involves continuous effort, time, and active individual engagement in coping with difficulties (Garmezy, 1991). In this context, resilience reflects individuals’ capacity to recover from setbacks, maintain psychological stability, and continue functioning effectively despite adversity. Accordingly, mental toughness is considered a fundamental personal resource that individuals must develop in order to achieve success and effectively cope with the challenges encountered in both everyday life and performance settings (Özsarı et al., 2021).

In sports environments, athletes frequently experience various psychological pressures while striving to achieve high levels of performance. Competitive demands, expectations from coaches and supporters, and the uncertainty of sporting outcomes often lead to psychological states such as anxiety, stress, and performance pressure (Mellalieu et al., 2009; La Fratta et al., 2021). The ability to effectively manage, regulate, and maintain psychological stability under such conditions is therefore crucial for athletes. Developing psychological competencies such as perseverance, determination, and self-discipline plays an essential role in helping athletes sustain performance during challenging moments of training and competition (Samur, 2018). Within this framework, mental toughness functions as a protective psychological factor that allows athletes to maintain motivation, cope with stress, and sustain performance levels over time.

Recent research further highlights the importance of mental toughness as a critical determinant of athletic success. For example, Aditya et al. (2024), in their study examining the effect of mental toughness on athletic performance, reported that resilience is among the most important psychological skills influencing athletes’ performance outcomes and contributes significantly to maintaining consistent performance levels during competitive situations. Because of its strong relationship with performance consistency and psychological stability, mental resilience has become one of the most widely studied constructs in sport psychology. As a result, it holds considerable importance for both athletes and coaches, particularly during high-pressure competition periods where psychological demands are most intense (Crust, 2004; Jones et al., 2007; Cowden, 2016; Jaeschke et al., 2016; Sorensen et al., 2016).

In the sport psychology literature, mental toughness is often conceptualized as a key component of psychological resilience. Jones et al. (2007) defined mental toughness as “a natural or developed psychological edge that enables athletes to cope better than their opponents with the many demands of competition, training, and performance-related situations, and that allows them to remain more focused, confident, and in control under pressure.” This definition emphasizes the capacity of mentally tough athletes to maintain task-oriented attention and emotional control despite challenging circumstances.

Similarly, Loehr (2002) argued that mentally tough athletes display a range of psychological responses that enable them to remain emotionally relaxed, calm, and resilient when facing adversity. According to this perspective, two key psychological competencies characterize mentally tough athletes. The first is the ability to generate and channel positive psychological energy during crises and demanding situations. The second is the capacity to adopt constructive and adaptive thinking patterns that support appropriate attitudes toward pressure, mistakes, and competitive challenges. These cognitive and emotional skills enable athletes to interpret stressful situations as opportunities for growth rather than threats to performance.

Mental toughness is therefore widely recognized as a central psychological attribute that enables athletes to sustain high levels of performance despite stress, fatigue, and competitive pressure (Gucciardi et al., 2015a,b). In football, where players are required to make rapid decisions while coping with physical and psychological demands, mental toughness plays a particularly important role. Mentally tough football players are better able to maintain concentration, regulate emotions, and execute tactical decisions effectively during high-pressure moments of the game (Crust and Clough, 2011). Moreover, athletes with higher levels of mental toughness demonstrate greater persistence, improved coping strategies, and enhanced performance stability during competition.

Previous studies also indicate that psychological training interventions can significantly enhance athletes’ mental toughness and resilience. Structured psychological skills training programs that incorporate techniques such as goal setting, imagery, and emotional regulation have been shown to strengthen athletes’ mental toughness, thereby improving decision-making abilities and maintaining performance consistency in competitive environments (Gucciardi et al., 2017). Consequently, developing mental toughness through systematic psychological training has become an important focus in modern sport psychology research and applied sport practice.

Although the terms mental toughness and mental toughness are sometimes used interchangeably in the sport psychology literature, they represent conceptually distinct constructs. Mental toughness is generally defined as a dynamic process reflecting an individual’s ability to adapt positively to adversity and recover from stress (Fletcher and Sarkar, 2012). In contrast, mental toughness is considered a relatively stable psychological trait that enables athletes to consistently perform at high levels despite pressure, challenges, and setbacks (Gucciardi et al., 2015a,b).

In the present study, mental toughness is operationalized through the Sport Mental Toughness Questionnaire (SMTQ), which captures dimensions such as confidence, control, and constancy. Therefore, while resilience provides a broader adaptive framework, the focus of this study is specifically on mental toughness as a performance-related psychological attribute.

### 1.2 Decision-making skill

Decision-making is one of the most fundamental cognitive characteristics that distinguishes humans from other living beings. It is generally defined as the process of selecting the most appropriate option among multiple available alternatives. This process is influenced by various internal factors such as motivation, stress, personality traits, prejudices, and intuition (Çolakkadıoğlu, 2013), as well as external factors including environmental conditions, social context, and cultural influences (Halis, 2002; Kurt, 2003). Because of these influences, decision-making is considered a complex cognitive and behavioral process that requires individuals to evaluate information, assess possible outcomes, and select the most appropriate course of action (Kahneman, 2011).

From a cognitive perspective, decision-making involves several sequential stages. First, individuals recognize the existence of a situation that requires a decision. Second, they interpret the situation and classify it appropriately based on prior knowledge and contextual cues. Third, individuals evaluate available alternatives and select the most suitable option. Finally, the selected decision is implemented through action (Yıldız, 2012). During this process, individuals often anticipate the potential consequences of their choices, which may increase psychological pressure and stress levels. If stress becomes excessively high during the decision-making phase, cognitive processing may become impaired, increasing the likelihood of incorrect or suboptimal decisions. Under such conditions, individuals may struggle to interpret situational cues accurately, evaluate alternatives effectively, and ultimately make appropriate decisions.

Decision-making processes play an important role not only in everyday life but also in performance-oriented domains such as sport. In sporting environments, the quality and accuracy of decisions can significantly influence performance outcomes. Accurate and timely decisions made during competition may contribute directly to success, whereas incorrect decisions can result in negative consequences and even competitive failure (Leveaux, 2010). Therefore, decision-making ability is widely considered a critical psychological and cognitive skill that athletes must develop in order to perform effectively in competitive situations.

Athletes frequently encounter dynamic and rapidly changing situations during competitions that require immediate and effective decision-making. In these situations, athletes who are able to quickly analyze the available information, review potential options, and evaluate the circumstances in a controlled and systematic manner are more likely to achieve successful performance outcomes. In contrast, athletes who experience difficulties in quickly and accurately evaluating game situations may struggle to produce effective responses and may therefore demonstrate lower performance levels (Tekin et al., 2009). Consequently, the ability to process information efficiently and make accurate decisions under time pressure is considered one of the key determinants of success in many sports.

In team sports such as football, decision-making plays an even more crucial role due to the dynamic, unpredictable, and time-constrained nature of the game. Football players must continuously interpret changing game situations, anticipate opponents’ actions, and select appropriate responses while operating under limited time and information constraints (Jones et al., 2009). This process requires players to perceive relevant environmental cues, evaluate alternative actions, and rapidly execute the most effective response (Williams and Ford, 2008). Because football is characterized by continuous interaction between teammates, opponents, and environmental constraints, players must repeatedly make tactical decisions throughout the match.

Previous research has highlighted that expert football players demonstrate superior perceptual-cognitive abilities compared to less experienced players. These athletes are more effective in recognizing patterns of play, anticipating opponents’ movements, and selecting appropriate tactical actions under pressure (Williams and Ward, 2007). As a result, decision-making ability, together with technical and tactical competencies, is widely considered one of the fundamental components of successful football performance (Vaeyens et al., 2007). Developing athletes’ decision-making skills through both training and psychological preparation is therefore an important objective in modern football development programs.

### 1.3 Tactical skills

Tactics can generally be defined as a set of short-term, practical actions used to achieve broader strategic objectives. In sports contexts, tactics refer to the application of strategies that aim to optimize performance through effective decision-making, situational awareness, and behavioral regulation during competition (Wein, 2004; Hughes and Franks, 2004). Tactical approaches guide how players respond to dynamic game situations and coordinate their actions with teammates in order to maximize competitive advantage. In this sense, tactics represent the operational dimension of strategy, translating long-term strategic goals into immediate and context-specific actions during gameplay.

In football and other team sports, tactics encompass a wide range of in-game behaviors and strategic practices. These include positioning, tempo management, spatial organization, marking opponents, pressing strategies, and offensive and defensive formations that collectively contribute to achieving success during competition. Tactical skills therefore involve the ability of players to interpret game situations, coordinate movements with teammates, and adapt their actions according to constantly changing environmental conditions. According to McPherson (2000), tactical skills can be defined as athletes’ capacity to analyze environmental conditions and team dynamics during the game, make appropriate decisions, and demonstrate optimal strategic behaviors in response to these situations.

From a cognitive perspective, tactical skills involve the integration of several perceptual and decision-making processes. Gréhaigne et al. (1997) describe tactical competence as the combination of multiple cognitive elements, including game intelligence, environmental awareness, rapid and accurate decision-making, strategic intuition, and the ability to adapt to evolving game contexts. These processes enable players to interpret complex game situations, anticipate opponents’ actions, and select the most appropriate tactical responses. As a result, tactical performance depends not only on physical abilities but also on athletes’ perceptual-cognitive capacities and their ability to process information effectively during competition.

In this context, football should not be viewed merely as a sport dominated by physical capacity. Rather, it represents a complex interactive system in which technical, tactical, cognitive, and psychological factors are closely interconnected (Williams and Reilly, 2000). Players must continuously interpret spatial and temporal information, adjust their positioning, and coordinate their actions with teammates to maintain effective team organization. These requirements make tactical understanding and decision-making central components of high-level football performance.

Recent developments in modern football further emphasize the growing importance of tactical competence in the game. The contemporary structure of football increasingly relies not only on individual technical skills but also on players’ ability to demonstrate game intelligence, process information rapidly, and execute tactical strategies effectively within a dynamic and unpredictable environment (Roca et al., 2012). In elite-level football, the ability to interpret game patterns, anticipate opponents’ behavior, and make rapid tactical adjustments often distinguishes successful teams and players from their competitors.

Furthermore, tactical skills are closely associated with perceptual-cognitive expertise, which allows players to recognize patterns of play and anticipate future events during matches (Memmert, 2015). Athletes with higher levels of tactical expertise are better able to adapt to changing game conditions, maintain effective positioning, and support team coordination throughout the match. Consequently, tactical competence has become one of the most important determinants of both individual and team performance in football.

Given the dynamic and interactive nature of the sport, the development of tactical skills has become a primary objective in contemporary football training programs. Training approaches that emphasize decision-making, spatial awareness, and game understanding are increasingly used to enhance players’ tactical abilities and overall performance (Sarmento et al., 2018). Therefore, tactical skills represent a multidimensional competence that integrates cognitive, psychological, and technical elements, all of which contribute significantly to successful football performance.

### 1.4 Psychological skills intervention programs

The concept of psychological skills has long been associated with individuals’ pursuit of high-level performance across various domains, including education, business, and sport (Vealey, 1988). Within the context of sport psychology, psychological skills refer to a set of mental abilities that enable athletes to regulate their thoughts, emotions, and behaviors in ways that support optimal performance. These skills typically include stress management, goal setting, self-confidence, mental toughness, attentional control, and emotional regulation (Holland et al., 2010). Because competitive sport environments are often characterized by intense pressure, uncertainty, and high expectations, the development of such psychological competencies is considered essential for achieving and maintaining high performance levels (Weinberg and Gould, 2019).

Psychological Skills Intervention (PSI) programs can be defined as the systematic and planned application of various psychological techniques designed to enhance athletes’ mental capacities and overall performance. These programs aim to increase athletes’ enjoyment of sport, support their ability to achieve high-level performance, reduce fear and anxiety in competitive environments, and enable them to cope effectively with pressure and stressful situations (Aksoy, 2024). PSI programs are typically structured interventions that incorporate a range of mental training techniques and are implemented through organized training sessions under the guidance of coaches or sport psychologists.

In recent years, psychological skills training has gained increasing attention within the field of sport psychology due to its demonstrated effectiveness in enhancing athletic performance and psychological wellbeing. Psychological skills training generally involves structured practices implemented within a specific training plan designed to support athletes in achieving optimal performance levels (Neff, 2010). These programs often include techniques such as goal setting, imagery, self-talk, attentional control, relaxation training, and emotional regulation strategies. By systematically applying these techniques, athletes can develop greater self-awareness and improve their ability to regulate their psychological states during both training and competition (Birrer and Morgan, 2010).

The primary goals of Psychological Skills Intervention (PSI) programs are to help athletes recognize their own abilities, develop awareness of their internal strengths, and enhance their self-confidence (Aktepe, 1999). In addition, these programs aim to strengthen athletes’ psychological skills and thereby improve overall performance outcomes (Gardner and Moore, 2004). Through repeated practice and guided psychological training, athletes learn how to manage stress effectively, maintain concentration, and sustain motivation during challenging situations.

Psychological skills training programs also play a crucial role in the development of several core psychological competencies necessary for high-level sport performance. These competencies include self-confidence, positive thinking, goal setting, concentration, motivation, attentional focus, and self-regulation abilities (Neff, 2010). The development of such competencies enables athletes to maintain emotional stability and cognitive clarity even under high-pressure competitive conditions.

Martens (1987) and Gould and Weinberg (2015) identified several fundamental psychological skills that are typically targeted in psychological skills training programs. These include imagery (visualization), stress management, goal setting, attentional and concentration skills, leadership development, self-talk strategies, coping with injury, and the development of self-confidence. Through systematic training and repeated application of these techniques, athletes can strengthen their psychological resilience and improve their ability to perform effectively in demanding sporting environments.

Overall, Psychological Skills Intervention (PSI) programs are increasingly recognized as an important component of modern sport training systems. By integrating psychological training with physical, technical, and tactical preparation, these programs contribute to the holistic development of athletes and support sustained performance success in competitive sport settings (Birrer et al., 2012).

### 1.5 The present study

Although previous research has examined the role of psychological skills training in enhancing athletic performance, relatively limited studies have investigated its simultaneous effects on multiple performance-related constructs such as mental toughness, decision-making, and tactical performance in football players, particularly within controlled experimental designs (Freitas et al., 2013; Vella-Fondacaro and Romano-Smith, 2023). Moreover, much of the existing literature tends to examine psychological outcomes and sport performance indicators separately rather than adopting a holistic perspective. Consequently, there is a growing need for integrative experimental studies that explore how psychological skills interventions influence both the cognitive and tactical components of football performance (Freitas et al., 2013; Ivarsson et al., 2020; Lochbaum et al., 2022). Such an approach may contribute to a deeper understanding of how psychological development translates into observable performance behaviors during competition.

The present study investigates the impact of Psychological Skills Intervention (PSI) programs on football players’ performance by focusing on three critical constructs: mental toughness, decision-making, and tactical skills. Previous literature indicates that PSI programs can enhance athletes’ psychological capacities and improve performance through techniques such as relaxation strategies, self-talk, imagery (visualization), attentional control, and goal setting (Weinberg and Gould, 2019; Vealey, 2007). These psychological strategies support athletes in managing stress, maintaining focus, and sustaining optimal performance during high-pressure competitive situations. In this context, psychological skills training is increasingly recognized as an essential component of comprehensive athlete development programs (Birrer and Morgan, 2010).

Mental toughness is defined as the ability to maintain psychological flexibility and resistance when facing stressful, demanding, or uncertain situations. Within the specific context of football, mental toughness plays a crucial role in influencing players’ decision-making accuracy, game perception, and the effective execution of tactical actions (Clough P. J. et al., 2002; Gucciardi et al., 2009a,b). Players with higher levels of mental toughness are better able to regulate their emotions, maintain concentration under pressure, and respond effectively to rapidly changing game situations. In this sense, mental toughness functions as an important psychological resource that supports cognitive processing and decision-making during competition.

Psychological Skills Intervention programs may enhance the speed and accuracy of football players’ decision-making processes by strengthening their mental toughness and cognitive control. Research in sport psychology suggests that psychological preparedness allows athletes to process environmental information more efficiently and respond more effectively to situational demands (Abernethy, 1991; Eccles and Tran, 2012). Improved decision-making abilities subsequently facilitate the effective application of tactical skills during gameplay. Tactical skills involve several key performance elements, including correct positioning, timing of actions, team coordination, and the practical implementation of strategic decisions on the field (Wein, 2004; Hughes and Franks, 2004). Therefore, decision-making can be considered a crucial link between psychological preparedness and tactical performance in football.

Recent studies continue to highlight the growing importance of psychological performance factors in sport. Contemporary research emphasizes that psychological skills such as attentional control, emotional regulation, and cognitive flexibility play a crucial role in optimizing athletic performance in dynamic environments (Birrer et al., 2012; Brown and Fletcher, 2017). Moreover, recent meta-analyses indicate that structured psychological interventions significantly improve both psychological and performance-related outcomes in athletes (Lochbaum et al., 2022).

Within this framework, the theoretical model proposed in this study suggests that Psychological Skills Intervention programs may influence football players’ tactical skills both directly and indirectly. Specifically, PSI programs are expected to strengthen mental toughness, which in turn enhances players’ decision-making abilities. Improved decision-making processes are then expected to facilitate more effective tactical behavior during matches. Thus, the model proposes that PSI programs may exert an indirect effect on tactical skills through mental toughness and decision-making while also potentially having a direct positive effect on tactical performance. This conceptual framework provides a comprehensive perspective highlighting the holistic contribution of PSI programs to football performance from psychological, cognitive, and practical viewpoints (Weinberg and Gould, 2019).

Empirical research examining PSI interventions in sport has provided important insights into the effectiveness of these programs. For example, Bordo et al. (2025) implemented an eight-week Psychological Skills Intervention program with athletes focusing on psychological competencies such as relaxation, attentional focus, and self-confidence development. The findings demonstrated that athletes in the intervention group experienced increased self-confidence, reduced anxiety levels, and improved refocusing abilities during performance situations. Similarly, Röthlin et al. (2020) applied a PSI program to experimental and control groups consisting of 95 athletes and reported that the intervention primarily enhanced the use of mental strategies such as self-talk and attentional focus.

In another study, Saputra et al. (2022) implemented a Psychological Skills Intervention program with football referees and reported improvements in mental toughness, decision-making skills, and referee observer evaluation scores. Lim et al. (2018) conducted a three-month PSI program with Paralympic table tennis players and observed significant improvements in psychological skills including self-talk, emotional control, and goal setting. Likewise, Vella-Fondacaro and Romano-Smith (2023) reported moderate to high improvements in mental toughness, competition anxiety, and coping skills among futsal players following a 10-session PSI program. Ballıkaya and Saraç (2024) also implemented a 12-week PSI program with high school volleyball players and reported significant improvements in coping strategies and performance-related psychological skills.

Research focusing specifically on football also provides valuable evidence regarding the effectiveness of psychological interventions. Orhan (2023) reported that an eight-week Psychological Skills Intervention program implemented with professional football players increased levels of self-talk, although it did not produce a statistically significant change in mental toughness. Additionally, Erdoğan Yüce et al. (2025) examined the effects of a 12-week PSI program on physiological and psychological variables among football players competing in the U16–U18 age categories. The results indicated positive effects on heart rate variability, agility, reaction time, and several psychological skills.

Similarly, Pepe et al. (2020) provided training focusing on mental toughness and decision-making skills to football players competing in the U19 category of professional teams. The findings indicated that the training program implemented with the experimental group positively influenced players’ competitive performance. Overall, the existing literature consistently demonstrates that PSI programs have beneficial effects on psychological variables across different sports contexts. However, many studies focusing specifically on football have primarily examined psychological variables or limited aspects of performance outcomes.

Given that football is a dynamic, time-sensitive, and cognitively demanding sport, the literature has not yet sufficiently explained how psychological skills and mental toughness translate into actual performance behaviors, nor through which mechanisms these psychological improvements influence tactical decision-making and in-game performance. Furthermore, many previous studies suffer from methodological limitations, including limited experimental control, lack of randomization, or reliance on single-dimensional measurement approaches. These limitations make it difficult to clearly determine the causal effects of Psychological Skills Intervention programs.

The innovative contribution of the present study lies in its systematic examination of how psychological skills interventions influence not only psychological outcomes but also the cognitive and tactical behaviors demonstrated by football players during performance situations. Within this framework, the study proposes that the development of psychological skills such as attentional focus, self-talk, emotional control, and psychological regulation enhances football players’ ability to make accurate and rapid decisions under competitive pressure. These improvements are expected to be reflected in higher levels of football performance indicators, including tactical awareness, correct positioning, and effective in-game coordination. Therefore, the present study offers an important contribution to the sport psychology literature at both theoretical and applied levels by addressing the mechanisms through which Psychological Skills Intervention programs influence football performance. In this context, the study aims to fill an important gap in the literature by examining the effects of PSI programs on football players’ mental toughness, decision-making, and tactical skills using a randomized controlled experimental design. Accordingly, the central research question of the study is formulated as follows: Do Psychological Skills Intervention programs implemented over an eight-week period affect the mental toughness, decision-making abilities, and tactical skills of football players training within professional football teams?

Based on this framework, the following hypotheses are proposed:

*H1:* The eight-week Psychological Skills Intervention (PSI) program applied to football players has a positive effect on their mental toughness.

*H2:* The eight-week Psychological Skills Intervention (PSI) program applied to football players has a positive effect on their decision-making skills.

*H3:* The eight-week Psychological Skills Intervention (PSI) program applied to football players has a positive effect on their tactical skills.

### 1.6 Conceptual model and theoretical framework

This study is grounded in three complementary theoretical perspectives that explain how Psychological Skills Intervention (PSI) programs may influence football players’ mental toughness, decision-making processes, and tactical performance. Integrating frameworks from sport psychology and sport cognition allows the conceptual model of the study to explain both the psychological mechanisms and the cognitive processes through which structured mental training contributes to athletic performance.

First, the study is theoretically based on the Psychological Skills Intervention (PSI) model, which posits that psychological competencies such as attentional focus, imagery, goal setting, self-talk, and emotional regulation can be systematically developed through structured training programs. These psychological skills are considered trainable performance factors that can significantly enhance athletes’ competitive functioning and performance consistency (Weinberg and Gould, 2019). PSI programs aim to strengthen athletes’ self-regulation abilities, enabling them to manage stress, maintain concentration, and regulate emotions during high-pressure situations frequently encountered in competitive sport (Birrer and Morgan, 2010; Vealey, 2007). Within sport psychology, psychological skills training has been shown to positively influence athletes’ confidence, concentration, and emotional control, which are essential determinants of performance outcomes (Hardy et al., 1996; Tod et al., 2011). Furthermore, PSI programs are increasingly integrated into modern football training environments because they support athletes’ ability to sustain optimal performance levels under psychological pressure while enhancing self-efficacy and mental preparedness (Gucciardi et al., 2009a,b). The fundamental premise of PSI programs is therefore that mental skills are teachable and that their systematic application within a sports environment can optimize athletes’ performance.

Second, the mental toughness dimension of the study is grounded in the 4Cs Model (Control, Commitment, Challenge, and Confidence) developed by Clough P. et al. (2002). This model conceptualizes mental toughness as a multidimensional construct that determines how effectively individuals cope with pressure, adversity, and competitive demands. Athletes who demonstrate higher levels of control are able to regulate emotions and maintain composure in stressful situations, while commitment reflects persistence and goal-oriented behavior during training and competition. The challenge component represents the perception of difficult situations as opportunities for growth, whereas confidence refers to athletes’ belief in their abilities and interpersonal influence (Clough P. J. et al., 2002).

Empirical research suggests that psychological skills training programs can enhance these dimensions of mental toughness by strengthening athletes’ coping strategies and psychological adaptability (Gucciardi et al., 2017; Nicholls et al., 2009). Increased mental toughness has been associated with improved performance consistency, greater persistence under pressure, and enhanced psychological recovery following mistakes during competition (Cowden et al., 2016). Within the context of football, mental toughness enables players to maintain tactical discipline, sustain motivation, and make effective decisions despite the dynamic and stressful nature of the game.

Third, the decision-making and tactical performance of football players are explained through the perceptual cognitive expertise approach within the sport cognition literature. This framework suggests that expert athletes develop superior abilities in processing environmental information, recognizing patterns of play, and selecting appropriate actions in complex and time-constrained situations (Williams and Ward, 2007). Decision-making in sport is therefore not solely a technical ability but also a cognitive process involving perceptual cue utilization, anticipation, attentional control, and situational awareness (Abernethy et al., 2005; Ward et al., 2002).

Football players are required to continuously interpret rapidly changing environmental cues such as teammates’ positioning, opponents’ movements, and available tactical spaces. Psychological skills interventions, particularly techniques aimed at improving attention regulation, imagery, and cognitive control, can strengthen these perceptual–cognitive mechanisms (Williams et al., 2011). For example, attentional training enhances players’ ability to focus on relevant cues while filtering out distractions, which contributes to faster and more accurate tactical decisions during match situations (Moran, 2012). Similarly, imagery training can improve anticipation and tactical visualization, allowing players to mentally rehearse game situations and optimize their responses during competition (Cumming and Williams, 2013).

When these three theoretical frameworks are considered together, they provide a holistic perspective suggesting that psychological skills interventions influence football performance through multiple interacting mechanisms. PSI programs enhance athletes’ self-regulation capacities and emotional control, which contribute to the development of mental toughness as conceptualized by the 4Cs model. At the same time, improvements in attentional control and cognitive processing support perceptual–cognitive expertise, thereby strengthening decision-making efficiency and tactical accuracy on the field.

Accordingly, the conceptual model of the present study proposes that Psychological Skills Intervention programs contribute to improved football performance by simultaneously enhancing psychological resilience and perceptual–cognitive decision-making processes. Through this integrated mechanism, PSI programs are expected to strengthen players’ capacity to cope with competitive pressure while also improving the cognitive accuracy required for tactical execution during matches.

## 2 Methods

### 2.1 Study sample size determination

To determine the required sample size for this study, an *a priori* power analysis was conducted using a two-group, three-time-point repeated measures MANOVA design. The analysis was performed based on Pillai’s trace statistic and the O’Brien–Shieh algorithm, which provides a correction for degrees of freedom and is recommended for multivariate repeated measures models. The calculations assumed a moderate within-subject effect size (*f* = 0.30), a significance level of 5% (*α* = 0.05), and a minimum statistical power of 0.75 (Faul et al., 2007). The results of the power analysis indicated that approximately 50 participants (at least 25 in each group) would be sufficient to reliably detect the expected effect. Accordingly, the final sample size of the study was determined in line with these power analysis results.

Although the statistical power of the study was calculated as 0.75, it is slightly below the commonly recommended threshold of 0.80 for behavioral sciences (Cohen, 1988). Therefore, the possibility of Type II error should be considered when interpreting non-significant findings, and future studies with larger sample sizes are recommended to increase statistical power.

### 2.2 Participants

In this study, purposive sampling was employed to select participants who met specific criteria aligned with the research objectives. This non-probability sampling technique involves the intentional selection of individuals based on characteristics, expertise, or experience considered relevant to addressing the research question (Creswell, 2014). The sample of the study consisted of football players from the youth academies of professional football clubs competing in the Turkish Football Federation (TFF) Development Leagues.

In this study, a combination of purposive and convenience sampling methods was employed. Initially, purposive sampling was used to identify football players who met specific inclusion criteria relevant to the research objectives. Subsequently, convenience sampling was applied to recruit participants from accessible teams within the Turkish Football Federation Development Leagues. This combined approach allowed the inclusion of participants with relevant characteristics while maintaining feasibility in data collection (Creswell, 2014).

Inclusion criteria for the study were:

(i) being between 18 and 20 years of age,
(ii) actively competing in the U-19 category,
(iii) having at least 3 years of licensed football experience, and
(iv) providing voluntary informed consent to participate in the study.

Exclusion criteria for the study were:

(i) being outside the 18–20 age range,
(ii) competing in categories other than U-19,
(iii) having less than three years of licensed football experience,
(iv) declining to provide voluntary informed consent, and.
(v) being a football player competing in the U-19 category of local amateur football clubs.

This study employed convenience sampling, a non-probability sampling method. Convenience sampling involves the inclusion of participants who are readily accessible to the researcher and offers advantages in terms of time and cost efficiency. However, because participant selection in this method is not based on randomization, the representativeness of the sample for the target population is limited. Consequently, the generalizability of the findings is constrained, and the results were interpreted with this limitation in mind (Büyüköztürk et al., 2018; Creswell, 2014).

To ensure group equivalence, the aim was to include an equal number of athletes in both groups. Although the power analysis indicated that 25 players per team would be sufficient, efforts were made to include as many eligible participants as possible who met the inclusion criteria and provided voluntary informed consent at the beginning of the study. Consequently, 30 players from each team were included in the study.

It was determined that one of the football teams competing in the Turkish Football Federation (TFF) Development Leagues would serve as the control group, while the other team would constitute the experimental group. Although the study initially began with 30 players from each team, during the subsequent weeks, 2 players from the control group and 3 players from the experimental group withdrew from the study for various reasons (e.g., injury, discontinuation of training, or participation in competitions at a higher category). Consequently, the study was completed with 28 players in the control group and 27 players in the experimental group.

The inclusion and exclusion criteria were carefully defined to ensure sample homogeneity and internal validity. These criteria were established based on previous sport science research emphasizing the importance of participant consistency in intervention studies (Creswell, 2014).

### 2.3 Randomization procedure

This study employed a randomized controlled experimental design to objectively and causally evaluate the effect of the intervention. The planning, implementation, and reporting phases of the study were conducted in accordance with the principles outlined in the CONSORT (Consolidated Standards of Reporting Trials) 2010 guidelines (Schulz et al., 2010). The randomized design was implemented to reduce selection bias and ensure baseline equivalence between the groups. The football players included in the study were recruited from two different professional youth teams competing in the Turkish Football Federation (TFF) Development Leagues.

To prevent contamination bias and preserve the natural structure of the training processes, randomization was implemented at the cluster level. Accordingly, the two teams participating in the study were assigned to the experimental and control groups using a computer-based random number generation method.^1^ The randomization procedure was conducted by an independent researcher who was not involved in the data collection or intervention implementation processes of the study. Prior to the completion of the assignment process, it was verified that the groups had comparable profiles in terms of age, sports experience, position distribution, and basic physical characteristics.

This approach is consistent with the principle of ensuring initial intergroup comparability, as emphasized in the CONSORT guidelines (Moher et al., 2010). Following randomization, the experimental group participated in an eight-week Psychological Skills Intervention (PSI) program, while the control group continued their routine football training program. Participant flow, including individuals who withdrew from the study and the final sample size included in the analyses, was reported in accordance with CONSORT flowchart principles. This methodological approach was adopted to enhance the internal validity of the study, minimize potential systematic errors, and strengthen the reliability of the findings.

It is important to acknowledge that the randomization process was conducted at the team (cluster) level rather than the individual level. This approach may introduce intra-cluster dependency, potentially violating the assumption of independence of observations. Although the current analyses were conducted at the individual level, future studies are recommended to apply multilevel modeling techniques or adjust for intra-cluster correlation coefficients (ICC) to more accurately estimate intervention effects (Hox et al., 2017). This limitation should be considered when interpreting the findings.

To ensure standardization in data collection, all measurement instruments were administered under similar environmental conditions and supervised by the researchers. Participants completed the scales individually in quiet settings to minimize external distractions. Data collection procedures were conducted at three time points (pre-test, mid-test, and post-test) to capture both short-term and longitudinal changes associated with the intervention.

Furthermore, all participants were informed about the confidentiality of their responses, and anonymity was maintained throughout the study to reduce potential response bias (Podsakoff et al., 2003).

**Figure 1:**
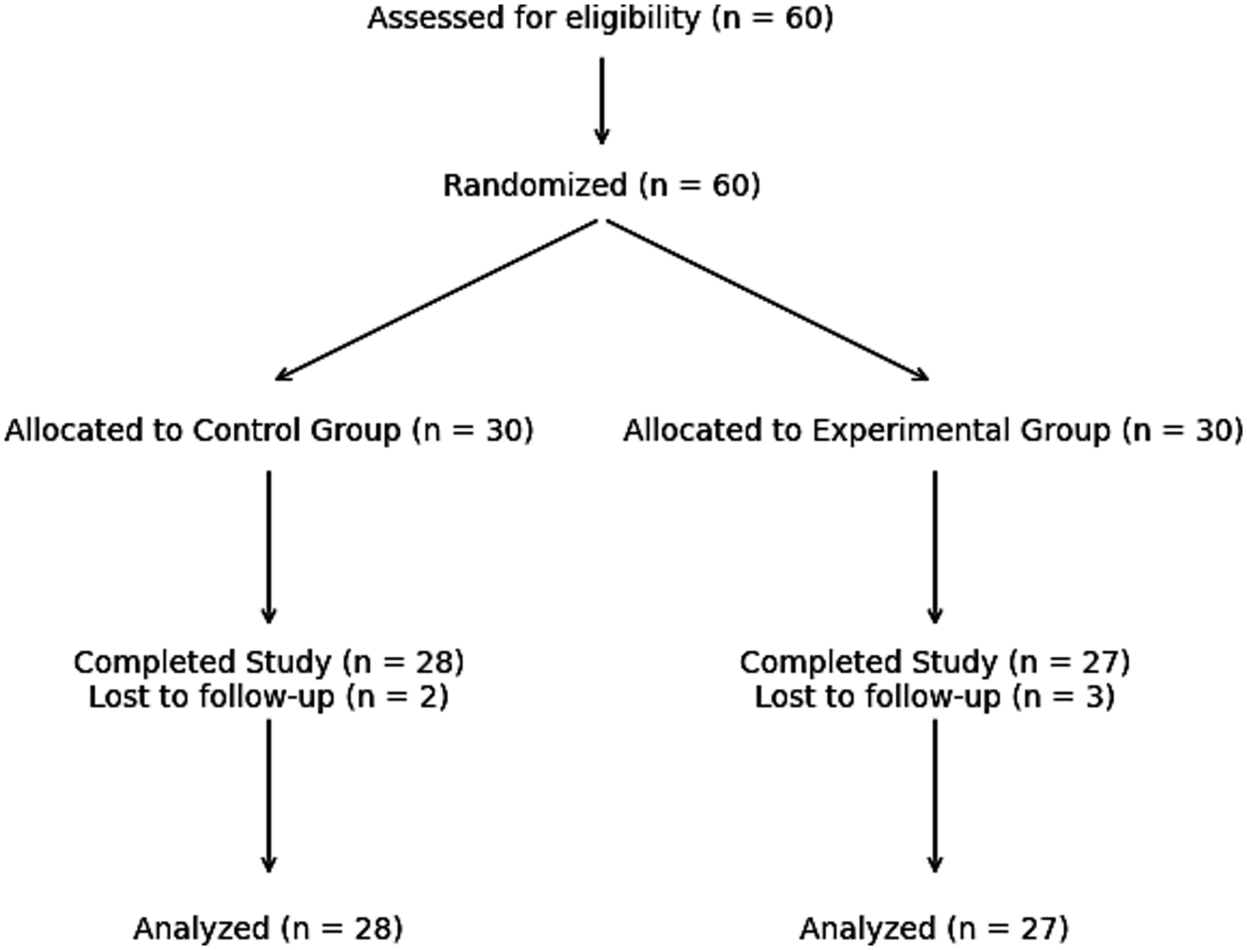
CONSORT flow diagram of participant recruitment, allocation, follow-up, and analysis.

The participant flow throughout the study, including recruitment, allocation, follow-up, and analysis phases, is presented in Figure 1 in accordance with CONSORT guidelines.

The study employed an experimental research design, specifically a pre-test–post-test control group model. In this design, measurements were conducted for both groups before and after the intervention; however, the training program was implemented only for the experimental group (Karasar, 2010). True experimental designs are considered to have the highest level of scientific rigor. The common characteristics of true experimental models include the use of multiple groups and the formation of these groups through random assignment. Accordingly, each study typically includes at least one experimental group and one control group, which are assumed to be equivalent in terms of other control variables (Creswell, 2014).

The present study employed a randomized controlled experimental design with repeated measures (pre-test, mid-test, and post-test). This design was selected to allow for the examination of both within-group changes over time and between-group differences attributable to the intervention. Randomized controlled designs are considered the gold standard for establishing causal relationships in intervention-based research (Hariton and Locascio, 2018).

Repeated measures ANOVA was preferred as the primary analytical technique because it allows for the evaluation of temporal changes within the same participants while also testing interaction effects between time and group variables. Additionally, independent samples t-tests were used to examine baseline equivalence between groups. The use of Bonferroni correction helped control for Type I error in multiple comparisons (Field, 2018).

### 2.4 Data collection tools

Participants in the study were asked to complete a socio-demographic information form prepared by the researchers, as well as the Sports Mental toughness Questionnaire, the Effective Decision-Making in Sport Scale, and the Tactical Skills Inventory for Sports, all of which are established measurement instruments available in the literature.

#### 2.4.1 Socio-demographic information form

The socio-demographic information form prepared by the researchers consisted of eight questions related to age, body height, body weight, body mass index (BMI), playing position, years of football experience, professional contract status, and the presence of superstitious beliefs. The descriptive statistics of the football players’ physical characteristics, including age, body height, body weight, and body mass index, are presented in Table 1. Additionally, the findings related to years of football experience, playing position, professional contract status, and superstitious beliefs are presented in Table 2.

**Table 1:** Descriptive statistics of participants according to their physical characteristics.

Variables	*n*	Control group			*n*	Experimental group		
		Min	Max	X ± SD		Min	Max	X ± SD
Age (yr)	28	18.00	19.00	18.82 ± 0.39	27	18.00	19.00	18.81 ± 0.40
Body height (m)	28	1.69	1.91	1.78 ± 0.07	27	1.70	1.94	1.79 ± 0.06
Body weight (kg)	28	60.00	76.00	67.21 ± 5.50	27	60.00	79.00	67.41 ± 0.5.04
BMI (kg/m^2^)	28	19.81	22.09	20.96 ± 0.55	27	19.37	22.53	20.99 ± 0.76

**Table 2:** The sociodemographic characteristics of groups.

	Variable	Control group		Experimental group	
		*n*	%	*n*	%
Playing football period (yr)	3–5	7	25.00	6	22.30
	6–8	15	53.60	11	40.70
	9 yıl ve üzeri	6	21.40	10	37.00
Position	Goal Keeper	4	14.30	3	11.20
	Defender	8	28.60	8	29.60
	Mid-Fielder	9	32.10	8	29.60
	Attacker	7	25.00	8	29.60
Having a professional contract	Evet	8	28.60	7	25.90
	Hayır	20	71.40	20	74.10
Having a superstition	Evet	10	35.70	8	40.70
	Hayır	18	64.30	16	59.30
Total		28	100	27	100

When Table 1 examined, it was determined that the average age of the football players in the control group was 18.82 ± 0.39 years, their average body height was 1.78 ± 0.07 m, their average body weight was 67.21 ± 5.50 kg and their average BMI was 20.96 ± 0.55 kg/m^2^.

The average age of the football players in the experimental group was 18.81 ± 0.40 years, their average body height was 1.79 ± 0.06 m, their average body weight was 67.41 ± 0.5.04 kg and their average BMI was 20.99 ± 0.76 kg/m^2^.

As presented in Table 2, according to the variable of years of playing football, 25% of the football players in the control group reported 3–5 years of experience, 53.6% reported 6–8 years, and 21.4% reported 9 years or more. Regarding playing positions, 14.3% of the players were goalkeepers, 28.6% were defenders, 32.1% were midfielders, and 25% were attackers. In terms of having a professional contract, 28.6% of the players reported having a contract, whereas 71.4% reported not having one. Furthermore, regarding superstitious beliefs, 35.7% of the players reported having such beliefs, while 64.3% reported not having them.

For the experimental group, 22.3% of the football players reported 3–5 years of playing experience, 40.7% reported 6–8 years, and 37% reported 9 years or more. With respect to playing positions, 11.2% were goalkeepers, 29.6% were defenders, 29.6% were midfielders, and 29.6% were attackers. Regarding professional contract status, 25.9% of the players reported having a professional contract, while 74.1% reported not having one. Additionally, 40.7% of the players reported having superstitious beliefs, whereas 59.3% reported not having such beliefs.

#### 2.4.2 Sport mental toughness questionnaire-SMTQ-14

The scale was developed by Sheard et al. (2009) to measure the mental toughness levels of athletes participating in sports. The scale consists of 14 items and three sub-dimensions. Each item is rated on a 4-point Likert scale. The sub-dimensions of the scale are as follows:

Confidence: Belief in one’s abilities and capacity to succeed in challenging situations, as well as the perception of personal competence and success (items 1, 5, 6, 11, 13, and 14).

Control: The ability to remain calm and composed without losing emotional control under intense stress and pressure (items 2, 4, 7, and 9).

Constancy: The ability to take responsibility and persist with determination in the pursuit of success (items 3, 8, 10, and 12).

The validity and reliability of the scale for Turkish athletes were established by Altıntaş and Koruç (2016). The Cronbach’s alpha coefficients reported for the sub-dimensions were 0.84 for Confidence, 0.51 for Constancy, and 0.79 for Control.

However, it should be noted that the Cronbach’s alpha coefficient for the Constancy sub-dimension (0.51) is below the generally accepted threshold for internal consistency. Although this value is consistent with previous validation studies, it may indicate limited reliability for this subscale within the current sample. Therefore, findings related to the Constancy dimension should be interpreted with caution (Taber, 2018).

#### 2.4.3 The scale of effective decision-making in sport (SEDMS)

This scale was developed by Çetin and Kara (2024) to measure the effectiveness of decision-making among athletes aged 18 and over who actively participate in sports. The scale consists of 15 items and two sub-dimensions. Each item is rated on a 5-point Likert scale.

The sub-dimensions of the scale are as follows:

Internal Decision-Making: Refers to athletes’ ability to generate alternative options and make decisions independently without being influenced by situational dynamics (items 1–7).

External Decision-Making: Refers to the influence of external factors that athletes may struggle with when making decisions (items 8–15).

The Cronbach’s alpha coefficients reported for the sub-dimensions were 0.85 for the Internal Decision-Making sub-dimension and 0.87 for the External Decision-Making sub-dimension.

#### 2.4.4 Tactical skills inventory for sports (TACSIS)

The scale was developed by Elferink-Gemser et al. (2004) to assess tactical skills in invasion games such as football, basketball, handball, rugby, and ice hockey. The scale consists of 22 items and four sub-dimensions. Each item is rated on a 6-point Likert scale.

The sub-dimensions of the scale are as follows:

- Positioning and Deciding: Refers to tactical skills related to taking appropriate positions and making effective decisions when in possession of the ball (items 1–9).
- Knowing About Ball Action: Refers to tactical skills associated with understanding and managing ball-related actions while in possession of the ball (items 10–13).
- Knowing About Others: Refers to tactical skills related to interpreting and anticipating opponents’ movements in off-ball situations (items 14–18).
- Acting in Changing Situations: Refers to tactical skills associated with adapting to different off-ball situations and responding effectively to dynamic game conditions (items 19–22).

The validity and reliability of the scale for Turkish athletes were established by Yarayan et al. (2019). The Cronbach’s alpha coefficients reported for the sub-dimensions of the scale were 0.88 for the Positioning and Deciding sub-dimension, 0.82 for the Knowing About Ball Action sub-dimension, 0.78 for the Knowing About Others sub-dimension, and 0.70 for the Acting in Changing Situations sub-dimension. Within the inventory, sub-dimensions 1 and 2 assess tactical skills related to offensive situations when the athlete is in possession of the ball, whereas sub-dimensions 3 and 4 evaluate tactical skills related to defensive situations when the athlete is without the ball.

As presented in Table 3, the Cronbach’s alpha values indicate that the internal consistency coefficients of the scales range between 0.76 and 0.95. Cronbach’s alpha is a reliability coefficient used to assess the internal consistency of multi-item scales. In other words, it evaluates the extent to which the items within a scale are interrelated and measure the same underlying construct. The coefficient ranges between 0 and 1, and a value of 0.70 or higher is generally considered to indicate an acceptable level of internal consistency (Nunnally and Bernstein, 1994). Therefore, these values suggest that the data obtained from the participants through these scales demonstrate an acceptable level of internal consistency.

**Table 3:** Descriptive values of the sub-dimensions of the scales.

Scales	Scale of sub-dimension	Test	Items	Cronbach Alpha	
				Control	Experimental
Mental toughness	Confidence	Pre	6	0.95	0.93
		Mid	6	0.95	0.90
		Post	6	0.93	0.90
	Control	Pre	4	0.92	0.90
		Mid	4	0.92	0.88
		Post	4	0.91	0.92
	Constancy	Pre	4	0.90	0.84
		Mid	4	0.90	0.86
		Post	4	0.86	0.85
Decision-making	Internal	Pre	7	0.89	0.87
		Mid	7	0.93	0.88
		Post	7	0.96	0.89
	External	Pre	8	0.82	0.74
		Mid	8	0.90	0.77
		Post	8	0.81	0.84
Tactical skills	Attacking	Pre	13	0.96	0.95
		Mid	13	0.95	0.92
		Post	13	0.95	0.91
	Positioning and Deciding	Pre	9	0.97	0.97
		Mid	9	0.97	0.96
		Post	9	0.97	0.95
	Knowing About Ball Action	Pre	4	0.97	0.96
		Mid	4	0.97	0.93
		Post	4	0.95	0.93
	Defending	Pre	9	0.92	0.90
		Mid	9	0.87	0.77
		Post	9	0.92	0.86
	Knowing About Other	Pre	5	0.92	0.92
		Mid	5	0.89	0.84
		Post	5	0.93	0.86
	Acting in Changing Situation	Pre	4	0.97	0.95
		Mid	4	0.83	0.76
		Post	4	0.89	0.91

### 2.5 Preparation of exercise program

In the study, football players in both the control and experimental groups regularly participated in football development training sessions prepared by the coaches of their respective clubs, and attendance at these sessions was mandatory for all players throughout the study period. In addition to this routine training program, 30 football players in the experimental group participated in a Psychological Skills Intervention (PSI) program totaling 16 h, consisting of 45-min sessions conducted twice per week over an eight-week period. The PSI program sessions were implemented at the Faculty of Sports Sciences at Uşak University. The PSI program followed the training, acquisition, and application phases described by Weinberg and Gould (2015).

During the PSI program, participants were provided with training on breathing exercises, progressive relaxation techniques, positive self-talk, imagery (visualization), and autogenic exercises. In addition, various instructional materials such as videos, slides, and visual aids were used throughout the sessions. A total of 27 football players in the experimental group successfully completed the PSI program. The 30 football players initially assigned to the control group did not participate in any PSI training program, and the control group completed the study with 28 players.

To enhance reproducibility, the PSI program was structured progressively across the eight-week period. The first 2 weeks focused on basic psychological awareness and relaxation techniques (e.g., breathing exercises and progressive muscle relaxation). Weeks three to five emphasized cognitive strategies such as self-talk and imagery training. The final weeks (six to eight) involved applied integration, where athletes practiced these skills in simulated sport-specific scenarios.

Intervention fidelity was monitored through session attendance records and direct supervision by the researchers. Participant adherence was ensured by requiring full attendance and active participation during each session. This structured progression aligns with established psychological skills training frameworks (Weinberg and Gould, 2019). The structure of the exercise program is presented in Table 3.

As presented in Table 4, the football players in the experimental group participated in 16 exercise sessions (Vealey, 2007), over an eight-week period, with each session lasting 45 min and conducted twice per week.

**Table 4:** Psychological skills intervention program.

Weeks	Time	Monday	Wednesday
1st week	45 min	Meeting. Importance of PST education. Applying Scales as Pre-test	Information about the study, pre-test application. Information about the Education process and application of the question and answer technique
2nd week	45 min	Decision, Decision-making. Decision-making theories	Decision making skills. The importance of decision making skills in football
3rd week	45 min	Breathing exercise, its benefits and its place in football player performance	Breathing practice
4th week	45 min	Gradual relaxation exercise, its benefits and its place in football player performance	Gradual relaxation practice
5 th week	45 min	Positive self-talk, its benefits and importance in football player performance	Positive self-talk practice. Applying scales as Mid-test
6th week	45 min	Imagery, its benefits and importance in football player performance	Imagery practice
7th week	45 min	Autogenic exercises, their benefits and their importance in football player performance	Autogenic exercises practice
8th week	45 min	Before, during and after the competition, the use and importance of techniques	Obtaining opinions and ideas about the techniques applied. Applying Scales as Post-test

### 2.6 Procedure

As the first step of the research process, permission was obtained from the football clubs to conduct the study. The players were invited to the meeting rooms of their respective clubs and were informed about the purpose and procedures of the study. Following this briefing, verbal consent was obtained from the football players, and they were asked to sign an informed consent form. A total of 30 football players from each club who signed the informed consent form agreed to participate in the study.

In addition, a socio-demographic information form, the Mental toughness Scale, the Effective Decision-Making in Sport Scale, and the Tactical Skills Inventory were administered as pre-test measurements to the football players in both the control and experimental groups. In the second phase of the study, the PSI program was implemented only with the football players in the experimental group, twice per week, with each session lasting 45 min. During the PSI intervention period, football players in both groups were required to continue attending their clubs’ regular conditioning training sessions.

After the fourth week of the study, the Mental Endurance Scale, the Effective Decision-Making in Sport Scale, and the Tactical Skills Inventory were re-administered as mid-test measurements. In the final phase of the study, following the completion of the eight-week intervention period, the Mental Endurance Scale, the Effective Decision-Making in Sport Scale, and the Tactical Skills Inventory were administered again as post-test measurements.

### 2.7 Data analysis

Scores obtained from the socio-demographic information form, the Sport Mental Toughness Questionnaire (SMTQ-14), the Scale of Effective Decision-Making in Sport (SEDMS), and the Tactical Skills Inventory for Sports (TACSIS) were entered into the SPSS 26 statistical software package developed by IBM for analysis. The normality of the data distribution was assessed by examining skewness and kurtosis values. The results of the normality tests for the participants are presented in Table 5.

**Table 5:** Normality test results of participants’ scale scores.

Scales	Sub-factors	Tests	Control group		Experimental group	
			Skewness	Kurtosis	Skewness	Kurtosis
Mental toughness	Confidence	Pre	−0.59	−1.25	−1.15	−0.07
		Mid	−0.59	−1.09	−1.07	−0.14
		Post	−0.57	−1.19	−0.96	0.35
	Control	Pre	−0.02	−1.13	0.33	−1.04
		Mid	−0.08	−1.14	0.33	−1.07
		Post	−0.02	−0.29	0.28	−0.90
	Constancy	Pre	−0.26	0.01	−0.08	−0.23
		Mid	−0.67	0.70	0.22	−0.49
		Post	0.01	0.85	0.17	−0.48
Decision making	Internal	Pre	−0.69	−1.24	−1.23	0.22
		Mid	−0.68	−1.29	−1.20	0.63
		Post	−0.67	−1.26	−0.91	0.84
	External	Pre	−0.71	−0.87	0.23	−1.08
		Mid	−0.65	−0.80	−0.43	−0.64
		Post	−0.47	−0.99	−1.09	1.40
Tactical skills	Attacking	Pre	−0.61	−0.37	−0.33	−0.21
		Mid	−0.60	−0.31	−0.25	0.46
		Post	−0.62	−0.40	0.27	−0.03
	Positioning and Deciding	Pre	−0.84	0.83	−0.79	1.19
		Mid	−0.89	1.17	−0.51	1.35
		Post	−0.87	0.78	−0.42	1.29
	Knowing About Ball Action	Pre	−0.04	−0.51	−0.06	−0.82
		Mid	−0.23	−0.23	−0.25	−0.48
		Post	0.34	−0.40	0.32	−0.62
	Defending	Pre	0.07	−1.30	−0.66	−0.19
		Mid	0.10	−1.21	−0.65	0.13
		Post	−0.16	−0.60	−1.26	1.99
	Knowing About Other	Pre	−0.15	−1.32	−0.64	−0.64
		Mid	−0.17	−1.19	−0.32	−1.23
		Post	−0.26	−0.61	−1.19	0.73
	Acting in Changing Situation	Pre	−0.22	−0.77	−0.98	0.11
		Mid	−0.05	−0.47	−1.26	1.21
		Post	−0.34	−0.13	−0.62	12

As presented in Table 5, the results of the normality analysis for the variables ranged between ±3. Previous studies in the literature suggest that skewness and kurtosis values within the range of ±1.5 (Tabachnick and Fidell, 2015) and ±2 (George and Mallery, 2016) can be considered acceptable indicators of normal distribution.

To examine differences between groups, the Independent Samples *t*-test was employed, as the sample consisted of two groups: experimental and control. In order to compare the pre-test, mid-test, and post-test scores obtained at three different time points, repeated measures analysis was conducted using analysis of variance (ANOVA). Additionally, a group × time interaction analysis was performed to examine the changes in the groups’ scores across the three measurement points. Effect sizes were interpreted using eta squared (*η*^2^) values in accordance with the criteria suggested by Cohen (1988) and Richardson (2011).

Furthermore, the Bonferroni *post hoc* test was applied to identify the source of significant differences among the measurements. In the present study, the group sizes were *n* = 28 for the control group and *n* = 27 for the experimental group. These sample sizes exceed the threshold at which overly conservative correction procedures, such as the Bonferroni adjustment, might substantially distort Type I error control (Abdi, 2007).

The assumption of sphericity for repeated measures ANOVA was tested using Mauchly’s test. In cases where the assumption was violated, Greenhouse–Geisser corrections were applied to adjust the degrees of freedom. These adjustments ensure more robust and reliable statistical inferences (Field, 2018).

## 3 Findings

Table 6 presents, the pre-test comparisons between the control and experimental groups revealed no statistically significant differences across any of the examined scales or sub-dimensions (*p* > 0.05). Specifically, no significant group differences were observed in the mental toughness sub-dimensions of confidence, control, and constancy. Similarly, the internal and external dimensions of effective decision-making did not differ significantly between the groups at baseline. With regard to tactical skills, both the attacking and defending dimensions, as well as the sub-dimensions related to positioning and decision-making, knowledge about ball action, knowledge about opponents, and acting in changing situations, showed comparable mean scores between the control and experimental groups. These findings indicate that the two groups were statistically equivalent prior to the intervention, supporting the assumption of baseline homogeneity and allowing subsequent post-test differences to be more confidently attributed to the experimental intervention rather than to pre-existing group differences.

**Table 6:** Pre-test comparison of control and experimental groups’ scores on mental toughness, decision-making, and tactical skills scales.

Scale	Sub-factors	Groups	*n*	M ± Sd	*t*	df	*p*
Mental toughness	Confidence	Control	28	16.03 ± 4.93	−0.73	53	0.47
		Experimental	27	16.92 ± 3.99			
	Control	Control	28	9.07 ± 2.78	0.65	53	0.52
		Experimental	27	8.59 ± 2.72			
	Constancy	Control	28	9.57 ± 0.74	−0.10	53	0.92
		Experimental	27	9.59 ± 0.80			
Effective decision making	Internal	Control	28	29.54 ± 3.88	−1.02	53	0.31
		Experimental	27	30.56 ± 3.52			
	External	Control	28	18.04 ± 2.03	0.22	53	0.83
		Experimental	27	17.93 ± 1.62			
Tactical skills	Attacking	Control	28	53.00 ± 8.24	−1.20	53	0.91
		Experimental	27	53.26 ± 7.77			
	Defending	Control	28	36.54 ± 6.67	−0.66	53	0.51
		Experimental	27	37.63 ± 5.56			
Attacking	Positioning and Deciding	Control	28	35.21 ± 5.55	−0.40	53	0.68
		Experimental	27	35.82 ± 5.46			
	Knowing About Ball Action	Control	28	17.79 ± 3.60	0.37	53	0.72
		Experimental	27	17.44 ± 3.30			
Defending	Knowing About Other	Control	28	20.50 ± 3.91	−0.63	53	0.53
		Experimental	27	21.15 ± 3.69			
	Acting in Changing Situation	Control	28	16.04 ± 3.81	−0.48	53	0.63
		Experimental	27	16.48 ± 2.98			

Table 7 presents the results of the repeated measures ANOVA, which indicated significant effects across several psychological variables. For Confidence, significant main effects of group (*F* = 69.68, *p* < 0.001, *η*^2^ = 0.62) and time (*F* = 18.05, *p* < 0.001, *η*^2^ = 0.25) were observed, along with a significant group × time interaction (*F* = 8.15, *p* = 0.001, *η*^2^ = 0.13). Bonferroni-adjusted comparisons indicated significantly higher post-test scores compared to pre-test scores in both groups (1 < 3), with additional improvements from the mid-test to the post-test in the experimental group (2 < 3).

**Table 7:** Comparison within the group for the mental toughness.

Variable	Group	Test	M ± Sd	df	Group			Time			Group*Time			Bon
					*F*	*p*	*η* ^2^	*F*	*p*	*η* ^2^	*F*	*p*	*η* ^2^	
Confidence	Control	Pre	16.93 ± 4.00	1.053	69.679	0.000**	0.621	18.052	0.000	0.254	8.149	0.001**	0.133	1 < 3
		Mid	17.26 ± 3.68											
		Post	10.67 ± 0.78											
	Experimental	Pre	16.04 ± 4.93	1.041	44.240	0.000**	0.621							1 < 3 2 < 3
		Mid	16.32 ± 4.85											
		Post	10.00 ± 0.54											
Control	Control	Pre	9.07 ± 2.77	1.027	4.533	0.051	0.144	19.227	0.000	0.266	3.748	0.056	0.066	–
		Mid	9.00 ± 2.78											
		Post	9.82 ± 2.65											
	Experimental	Pre	8.59 ± 2.72	1.070	15.145	0.000**	0.368							1 < 2 1 < 3 2 < 3
		Mid	9.00 ± 2.70											
		Post	10.70 ± 2.95											
Constancy	Control	Pre	9.57 ± 0.74	2	7.451	0.001**	0.216	34.087	0.000	0.391	7.626	0.001**	0.126	1 < 3
		Mid	9.75 ± 0.75											
		Post	10.000.54±											
	Experimental	Pre	9.59 ± 0.80	1.418	27.447	0.000**	0.514							1 < 3 2 < 3
		Mid	9.78 ± 0.64											
		Post	10.67 ± 0.78											

**p < 0.01, *η*^2^ = 01–0.05: Small effect, 06–0.13: Medium effect, 0.14 and above: Large effect, 1 = Pre Test, 2 = Mid Test, 3 = Post Test.

For Control, the main effect of group was not statistically significant (*F* = 4.53, *p* = 0.051, *η*^2^ = 0.14), whereas a significant main effect of time was observed (*F* = 19.23, *p* < 0.001, *η*^2^ = 0.27). The group × time interaction did not reach statistical significance (*F* = 3.75, *p* = 0.056, *η*^2^ = 0.07). However, post hoc analyses indicated a progressive increase in Control scores in the experimental group (1 < 2, 1 < 3, 2 < 3).

For Constancy, significant main effects of group (*F* = 7.45, *p* = 0.001, *η*^2^ = 0.22) and time (*F* = 34.09, *p* < 0.001, *η*^2^ = 0.39) were detected, along with a significant group × time interaction (*F* = 7.63, *p* = 0.001, *η*^2^ = 0.13). These results indicated higher post-test scores compared to pre-test scores in both groups (1 < 3), with additional improvements from the mid-test to the post-test in the experimental group (2 < 3).

**Figure 2:**
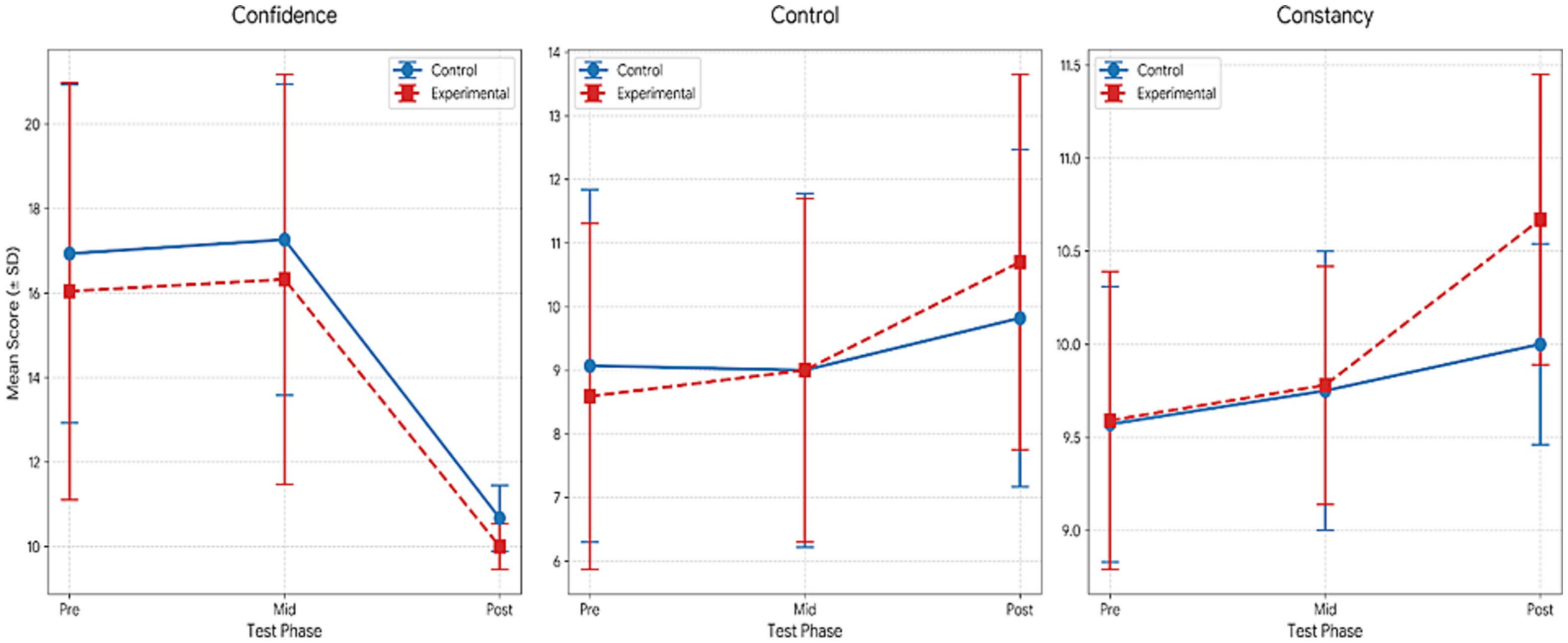
Comparison of confidence, control, and constancy mean scores between the control and experimental groups across pre-test, mid-test, and post-test phases.

These findings are visually supported by Figure 2, which illustrates the mean score trajectories of Confidence, Control, and Constancy for both the experimental and control groups across the pre-test, mid-test, and post-test measurement points.

**Figure 3:**
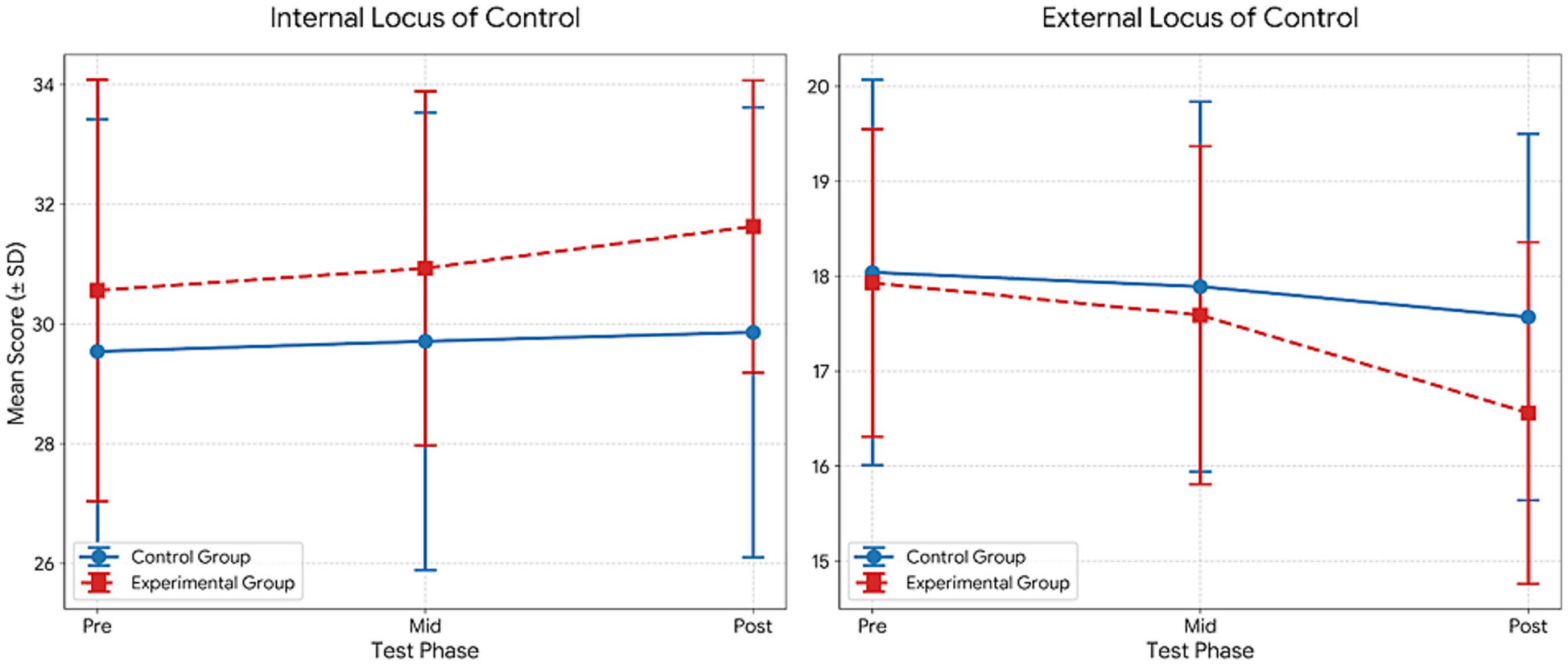
Comparison of mean scores for Internal and External Locus of Control between the experimental and control groups across pre-test, mid-test, and post-test phases.

Table 8 repeated measures ANOVA results demonstrated significant effects for Internal and External dimensions across groups and measurement times. For Internal, significant main effects of group (*F* = 8.62, *p* = 0.001, *η*^2^ = 0.24) and time (*F* = 15.50, *p* < 0.001, *η*^2^ = 0.23) were observed, together with a significant group × time interaction (*F* = 4.80, *p* = 0.010, *η*^2^ = 0.08). Bonferroni-adjusted post-hoc comparisons indicated significantly higher post-test scores compared to pre-test scores in both groups (1 < 3). At the same time, the experimental group also demonstrated significant increases from mid-test to post-test (2 < 3). For External, the analysis revealed significant main effects of group (*F* = 11.40, *p* < 0.001, *η*^2^ = 0.30) and time (*F* = 27.95, *p* < 0.001, *η*^2^ = 0.35), as well as a significant group × time interaction (*F* = 7.04, *p* = 0.003, *η*^2^ = 0.12). Post-hoc analyses showed significantly lower post-test scores compared to pre-test scores in both groups (1 < 3), with additional significant decreases from mid-test to post-test in the experimental group (2 < 3), indicating a more pronounced reduction in External scores over time in the experimental condition. These results are further illustrated in Figure 3, which presents the mean score changes for Internal and External Locus of Control across the pre-test, mid-test, and post-test measurements for both the experimental and control groups.

**Table 8:** Comparison within the group for effective decision-making.

Variable	Group	Test	M ± Sd	df	Group			Time			Group*Time			Bon
					*F*	*p*	*η* ^2^	*F*	*p*	*η* ^2^	*F*	*p*	*η* ^2^	
Internal	Control	Pre	29.54 ± 3.88	2	8.623	0.001**	0.242	15.495	0.000**	0.226	4.796	0.010*	0.083	1 < 3
		Mid	29.71 ± 3.82											
		Post	29.86 ± 3.76											
	Experimental	Pre	30.56 ± 3.52	1.258	9.968	0.002*	0.277							1 < 3 2 < 3
		Mid	30.93 ± 2.96											
		Post	31.63 ± 2.44											
External	Control	Pre	18.04 ± 2.03	1.489	11.400	0.000**	0.297	27.947	0.000**	0.345	7.037	0.003**	0.117	1 < 3
		Mid	17.89 ± 1.95											
		Post	17.57 ± 1.93											
	Experimental	Pre	17.93 ± 1.62	1.581	18.039	0.000**	0.410							1 < 2 1 < 3 2 < 3
		Mid	17.59 ± 1.78											
		Post	16.56 ± 1.80											

*p < 0.05, **p < 0.01, ***p < 0.001, *η*^2^ = 01–0.05: Small effect, 06–0.13: Medium effect, 0.14 and above: Large effect, 1 = Pre Test, 2 = Mid Test, 3 = Post Test.

**Figure 4:**
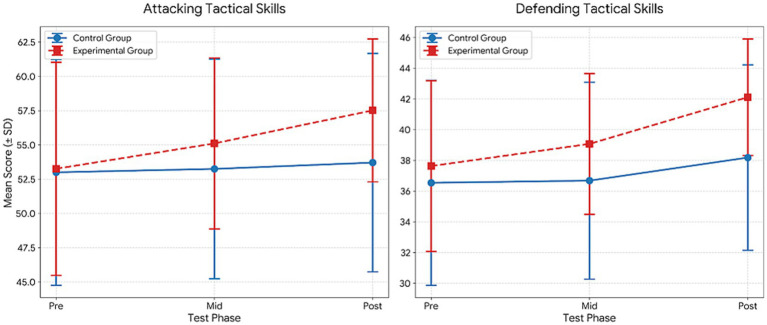
Comparison of mean scores for attacking and defending tactical skills between the experimental and control groups over three test phases (pre, mid, and post-test).

Table 9 repeated measures ANOVA revealed significant effects for Attacking and Defending performance variables across groups and measurement times. For Attacking, significant main effects of group (*F* = 19.91, *p* < 0.001, *η*^2^ = 0.42) and time (*F* = 28.71, *p* < 0.001, *η*^2^ = 0.35) were observed, along with a significant group × time interaction (*F* = 14.52, *p* < 0.001, *η*^2^ = 0.22). Bonferroni-adjusted post-hoc comparisons indicated significantly higher post-test scores compared to pre-test scores in both groups (1 < 3). In contrast, the experimental group demonstrated significant progressive improvements across all measurement points (1 < 2, 1 < 3, 2 < 3). For Defending, the analysis also showed a significant main effect of group (*F* = 5.60, *p* = 0.022, *η*^2^ = 0.17). A significant main effect of time (*F* = 27.85, *p* < 0.001, *η*^2^ = 0.35), accompanied by a substantial group × time interaction (*F* = 5.51, *p* < 0.005, *η*^2^ = 0.10). Post-hoc analyses revealed significantly higher post-test scores compared to pre-test scores in both groups (1 < 3), with the experimental group exhibiting a marked stepwise increase from pre-test to mid-test and from mid-test to post-test (1 < 2, 2 < 3), indicating stronger performance gains in Defending over time in the experimental condition. These results are further illustrated in Figure 4, which depicts the mean score trajectories for Attacking and Defending tactical performance across the pre-test, mid-test, and post-test phases for both the experimental and control groups.

**Table 9:** Comparison within the group for the tactical skills.

Variable	Group	Test	M ± Sd	df	Group			Time			Group*Time			Bon
					*F*	*p*	*η* ^2^	*F*	*p*	*η* ^2^	*F*	*p*	*η* ^2^	
Attacking	Control	Pre	53.00 ± 8.24	2	19.912	0.000**	0.424	28.710	0.000**	351	14.515	0.010*	0.215	1 < 3
		Mid	53.25 ± 8.01											
		Post	53.71 ± 7.97											
	Experimental	Pre	53.26 ± 7.77	1.163	9.968	0.000**	0.446							1 < 3 1 < 2 2 < 3
		Mid	55.11 ± 6.24											
		Post	57.52 ± 5.21											
Defending	Control	Pre	36.54 ± 6.67	1.089	5.600	0.022*	0.172	27.850	0.001**	0.345	5.507	0.005*	0.095	1 < 3
		Mid	36.68 ± 6.41											
		Post	38.18 ± 6.03											
	Experimental	Pre	37.63 ± 5.56	1.251	24.102	0.000**	0.481							1 < 2 1 < 3 2 < 3
		Mid	39.07 ± 4.58											
		Post	42.11 ± 3.79											

*p < 0.05, **p < 0.01, ***p < 0.001, *η*^2^ = 01–0.05: Small effect, 06–0.13: Medium effect, 0.14 and above: Large effect, 1 = Pre Test, 2 = Mid Test, 3 = Post Test.

**Figure 5:**
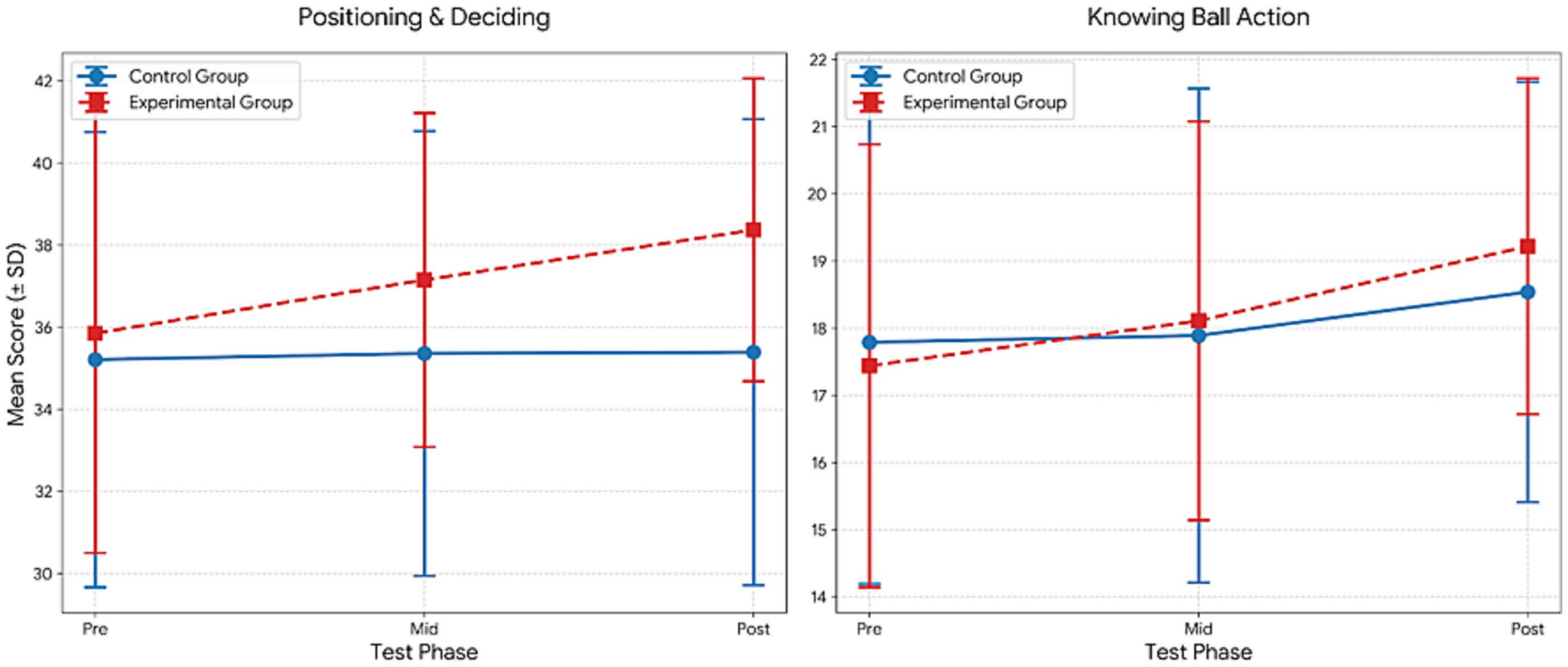
Developmental trajectories of attacking sub-skills: Positioning and Deciding (left) and Knowing About Ball Action (right). Data points represent means for the experimental and control groups across pre-test, mid-test, and post-test phases.

Table 10 repeated measures ANOVA results for Positioning, Deciding, and Knowing About Ball Action are presented. For Positioning and Deciding, the main effect of group was not statistically significant (*F* = 0.95, *p* = 0.377, *η*^2^ = 0.03). In contrast, a significant main effect of time was observed (*F* = 15.94, *p* < 0.001, *η*^2^ = 0.23). In addition, a significant group × time interaction emerged (*F* = 11.98, *p* < 0.001, *η*^2^ = 0.18), indicating differential changes over time between groups. Bonferroni-adjusted post-hoc comparisons showed no significant differences across measurement points in the control group, while the experimental group demonstrated significant stepwise increases from pre-test to mid-test and from mid-test to post-test (1 < 2, 1 < 3, 2 < 3). For Knowing About Ball Action, a significant main effect of group (*F* = 4.71, *p* = 0.028, *η*^2^ = 0.15) and a significant main effect of time (*F* = 18.16, *p* < 0.001, *η*^2^ = 0.26) were observed; however, the group × time interaction did not reach statistical significance (*F* = 2.87, *p* = 0.082, *η*^2^ = 0.05). Post-hoc analyses indicated significantly higher post-test scores compared to pre-test scores in both groups (1 < 3), with the experimental group additionally showing significant increases from pre-test to mid-test and from mid-test to post-test (1 < 2, 2 < 3). These findings are further illustrated in Figure 5, which displays the developmental trajectories of the attacking sub-skills Positioning, Deciding, and Knowing About Ball Action for both the experimental and control groups across the pre-test, mid-test, and post-test phases.

**Table 10:** Comparison within the group for attacking tactical skills.

Variable	Group	Test	M ± Sd	df	Group			Time			Group*Time			Bon
					*F*	*p*	*η* ^2^	*F*	*p*	*η* ^2^	*F*	*p*	*η* ^2^	
Positioning and Deciding	Control	Pre	35.21 ± 5.55	1.622	0.953	0.377	0.034	15.937	0.000**	0.231	11.978	0.000**	0.184	-
		Mid	35.36 ± 5.42											
		Post	35.39 ± 5.68											
	Experimental	Pre	35.85 ± 5.35	1.251	14.618	0.000**	0.360							1 < 3 1 < 2 2 < 3
		Mid	37.15 ± 4.07											
		Post	38.37 ± 3.69											
Knowing About Ball Action	Control	Pre	17.79 ± 3.60	1.315	4.713	028*	0.149	18.163	0.000**	0.255	2.867	0.082	0.051	1 < 3
		Mid	17.89 ± 3.68											
		Post	18.54 ± 3.13											
	Experimental	Pre	17.44 ± 3.30	1.226	13.848	0.000**	0.348							1 < 2 1 < 3 2 < 3
		Mid	18.11 ± 2.97											
		Post	19.22 ± 2.50											

*p < 0.05, **p < 0.01, ***p < 0.001, *η*^2^ = 01–0.05: Small effect, 06–0.13: Medium effect, 0.14 and above: Large effect, 1 = Pre Test, 2 = Mid Test, 3 = Post Test.

**Figure 6:**
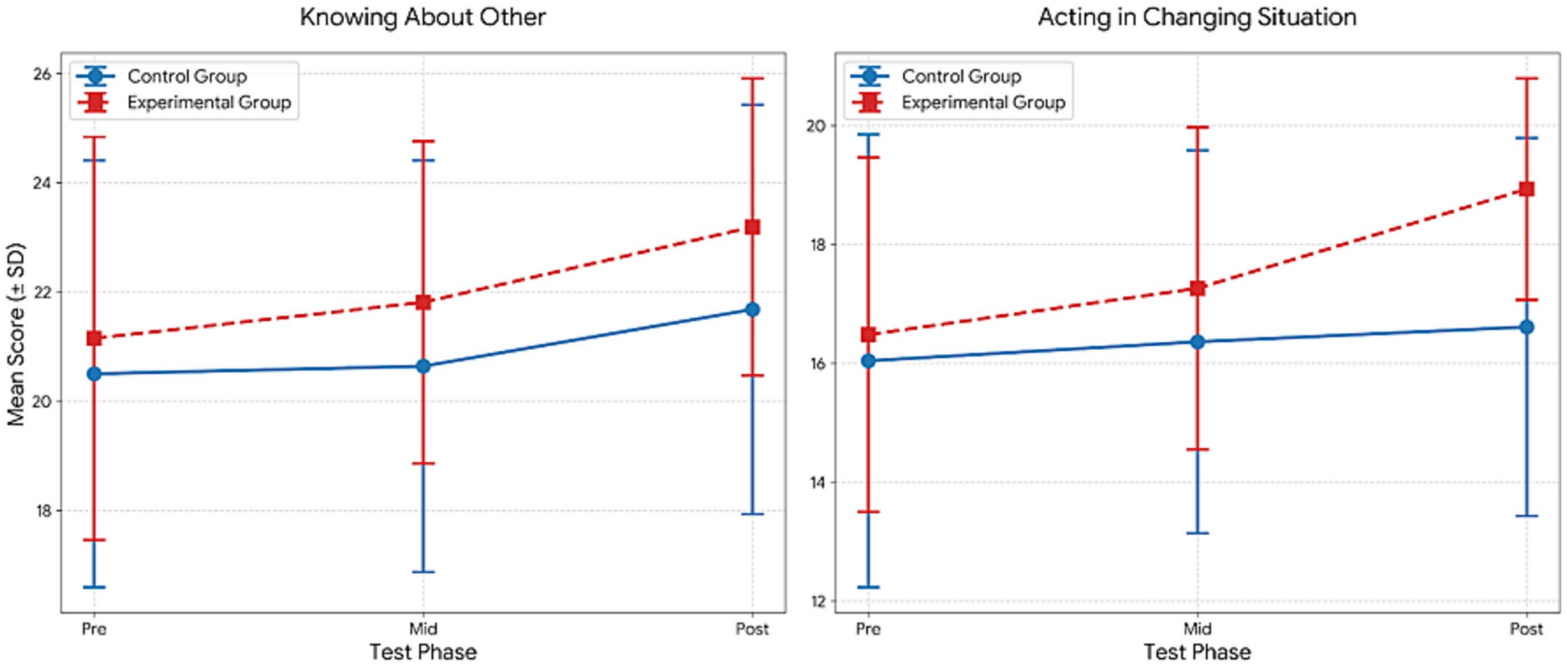
Developmental progress in defensive sub-skills: Knowing About Other (left) and Acting in Changing Situation (right). Mean scores are plotted for the experimental and control groups across pre-test, mid-test, and post-test phases.

Table 11 repeated measures ANOVA results for Knowing About Other and Acting in Changing Situation are presented. For Knowing About Other, significant main effects of group (*F* = 5.64, *p* = 0.023, *η*^2^ = 0.17) and time (*F* = 15.64, *p* < 0.001, *η*^2^ = 0.23) were observed, whereas the group × time interaction was not statistically significant (*F* = 1.05, *p* = 0.324, *η*^2^ = 0.02). Bonferroni-adjusted post-hoc comparisons indicated significantly higher post-test scores compared to pre-test scores in both groups (1 < 3), with the experimental group additionally showing significant increases from pre-test to post-test and from mid-test to post-test (1 < 3, 2 < 3). For Acting in Changing Situation, the main effect of group was not significant (*F* = 1.36, *p* = 0.266, *η*^2^ = 0.05), while a significant main effect of time was found (*F* = 17.69, *p* < 0.001, *η*^2^ = 0.25), together with a significant group × time interaction (*F* = 7.24, *p* = 0.002, *η*^2^ = 0.12). Post-hoc analyses revealed no significant differences across measurement points in the control group, whereas the experimental group demonstrated significant stepwise improvements from pre-test to mid-test and from mid-test to post-test (1 < 2, 1 < 3, 2 < 3). These results are further illustrated in Figure 6, which depicts the developmental progress of the defensive sub-skills Knowing About Other and Acting in Changing Situation for both the experimental and control groups across the pre-test, mid-test, and post-test phases.

**Table 11:** Comparison within the group for defending tactical skills.

Variable	Group	Test	M ± Sd	df	Group			Time			Group*Time			Bon
					*F*	*p*	*η* ^2^	*F*	*p*	*η* ^2^	*F*	*p*	*η* ^2^	
Knowing About Other	Control	Pre	20.50 ± 3.91	1.049	5.644	0.023*	0.173	15.641	0.000**	0.228	1.047	0.324	0.019	1 < 3
		Mid	20.64 ± 3.77											
		Post	21.68 ± 3.75											
	Experimental	Pre	21.15 ± 3.69	1.222	10.113	0.002**	0.280							1 < 3 2 < 3
		Mid	21.81 ± 2.95											
		Post	23.19 ± 2.72											
Acting in Changing Situation	Control	Pre	16.04 ± 3.81	2	1.357	0.266	0.048	17.692	0.000**	0.250	7.244	0.002**	0.120	-
		Mid	16.36 ± 3.22											
		Post	16.61 ± 3.18											
	Experimental	Pre	16.48 ± 2.98	1.367	21.801	0.000**	0.456							1 < 2 1 < 3 2 < 3
		Mid	17.26 ± 2.71											
		Post	18.93 ± 1.86											

*p < 0.05, **p < 0.01, ***p < 0.001, *η*^2^ = 01–0.05: Small effect, 06–0.13: Medium effect, 0.14 and above: Large effect, 1 = Pre Test, 2 = Mid Test, 3 = Post Test.

## 4 Discussion

This section interprets the findings of the present study in the context of the effects of Psychological Skills Intervention (PSI) programs on football players’ mental toughness, decision-making processes, and tactical skills. The results are discussed in relation to previous research in order to highlight both similarities and differences. Additionally, the potential contributions of PSI programs to on-field performance are evaluated. Finally, the limitations of the study and directions for future research are addressed, providing insights into the practical implications of the findings for applications in sport psychology.

The first finding of this study indicates that psychological skills intervention programs can have significant effects on the dimensions of mental toughness among football players. Previous systematic reviews have demonstrated that psychological skills training can enhance key components of mental toughness in athletes, including self-confidence, control, focus, and determination (Stamatis et al., 2020; Gucciardi et al., 2009a,b; Clough P. J. et al., 2002; Gucciardi et al., 2015a,b).

An alternative explanation for the decrease in self-confidence may be related to increased self-awareness following the intervention. As athletes become more cognitively engaged and reflective, they may evaluate their performance more critically, which can temporarily reduce perceived confidence levels (Beilock and Carr, 2001). Additionally, competitive pressure and performance expectations during the intervention period may have contributed to this unexpected outcome.

These findings are consistent with prior evidence in the sport psychology literature suggesting that psychological interventions contribute to the development of athletes’ mental toughness profiles (Ajilchi et al., 2022; Vella-Fondacaro and Romano-Smith, 2023; Gucciardi et al., 2008; Golby and Wood, 2016).

In the present study, significant increases were observed in the control and constancy sub-dimensions of mental toughness in the experimental group, whereas decreases in self-confidence scores were detected in both groups during the post-test period. In the literature, experimental studies examining the effects of PSI programs on the sub-dimensions of mental toughness in athletes have generally reported significant differences between pre-test and post-test measurements in the experimental group. For example, Karademir (2019), in a quasi-experimental study with young football players, reported that following an eight-week psychological skills training program, the confidence and control sub-dimension scores of the experimental group increased significantly from pre-test to post-test. In contrast, no significant change was observed in the control group.

Similarly, Şahin and Çelik (2024) reported that a six-week mental toughness development program implemented with basketball sports school students resulted in statistically significant increases in the control, constancy, and self-confidence sub-dimensions in the experimental group in pre-test–post-test comparisons. Furthermore, another experimental study conducted with futsal players demonstrated that a PSI program was effective in improving the confidence, consistency, and control dimensions in the experimental group (Lis et al., 2023). In contrast, Özer (2025) reported that although positive improvements were observed in overall mental toughness and its self-confidence sub-dimension among arm wrestlers following a PSI program, these improvements did not reach statistical significance in the pre-test–post-test comparisons.

Liew et al. (2019) emphasized that mental toughness plays a central role in athletes’ performance and adaptation processes, and that the development of these skills can positively influence decision-making and tactical performance. However, Urfa and Aşçı (2018) argue that the effectiveness of PSI programs may vary depending on factors such as the type of sport, the duration of the intervention, and the specific techniques employed. In this context, the findings obtained in the present study may be explained by the possibility that the PSI program had a limited direct effect on enhancing self-confidence. Additionally, contextual factors such as field conditions and the pressure associated with competition may have exerted suppressive effects on athletes’ self-confidence levels.

The second finding of this study indicates that both the control and experimental groups demonstrated increases over time in the internal decision-making sub-dimension; however, the increase observed in the experimental group was more consistent and pronounced. The significant group × time interaction suggests that the intervention was effective in enhancing internal decision-making processes. In contrast, a decrease was observed in the external decision-making sub-dimension in both groups, with a more pronounced decline in the experimental group. Moreover, the differences between all measurement time points were statistically significant, indicating that the intervention was effective in reducing the perception of external influences on decision-making. Overall, these findings suggest that the psychological skills intervention program improves athletes’ decision-making processes by strengthening their internal control mechanisms while reducing their reliance on external factors. Consistent with these results, the literature indicates that PSI programs enhance skills such as attentional control, self-awareness, internal regulation, and the ability to think effectively under pressure, enabling athletes to make faster and more accurate decisions during competition (Williams and Reilly, 2000; Tenenbaum and Bar-Eli, 1993).

In dynamic sports such as football, where decision-making occurs under conditions of time pressure, uncertainty, and constant interaction with opponents, strengthening internal orientation and reducing external distractions play a critical role (Bar-Eli et al., 2011). Accordingly, findings indicating that football players’ perceptions of internal control increased while their dependence on external factors decreased following the PSI program suggest that they exhibited more autonomous and consistent decision-making behaviors (Weinberg and Gould, 2019; Benabou and Tirole, 2003). Similarly, research conducted with handball players has shown that PSI programs can improve athletes’ decision-making processes and performance-related cognitive characteristics over time (Martiny et al., 2024).

Furthermore, PSI-type psychological skills training implemented with football referees who face a distinct dimension of decision-making demands on the field has also been shown to strengthen mental toughness and executive decision-making behaviors (Aksoy, 2024). PSI programs have been reported to exert significant effects not only on technical performance but also on athletes’ psychological and cognitive processes (Weinberg and Gould, 2019). Moreover, PSI programs are known to enhance participants’ intrinsic motivation while reducing external orientations, thereby strengthening cognitive performance and promoting more autonomous decision-making behaviors (Benabou and Tirole, 2003; Deci and Ryan, 2000; Ryan and Deci, 2017).

The findings of this study indicate that the PSI program enhanced football players’ ability to make more conscious and autonomous decisions based on their own knowledge, perceptions, and intrinsic motivation, while reducing their tendency to rely on environmental pressure and external factors when making decisions. This improvement may be attributed to athletes’ increased capacity to respond in a more controlled, consistent, and strategic manner when facing the rapid and uncertain situations commonly encountered during matches. With regard to the attacking and defending sub-dimensions of tactical skills, significant increases over time were observed in the experimental group, and the group × time interaction effects indicate that the implemented PSI program was effective. Specifically, significant improvements were found in both attacking and defending skills in the experimental group across the pre-test–mid-test, pre-test–post-test, and mid-test–post-test comparisons. In contrast, in the control group, only the pre-test–post-test comparison showed a statistically significant difference.

With regard to offensive tactical skills, the experimental group demonstrated significant and consistent increases over time in positioning and decision-making, as well as in understanding ball movement. Similarly, in terms of defensive tactical skills, the experimental group showed significant and consistent improvements over time in recognizing other players and responding to changing situations. The Bonferroni *post hoc* analyses indicated that the differences between all measurement time points were statistically significant within the experimental group. In contrast, the increases observed in the control group were limited. The significant group × time interaction effects suggest that the intervention program was more effective in enhancing these tactical skills in the experimental group compared to the control group.

In addition to sample-related limitations, potential sources of bias and confounding variables should be considered when interpreting the findings. Factors such as differences in coaching styles, training intensity, competitive exposure, and team dynamics may have influenced the observed outcomes independently of the intervention. Furthermore, the use of self-report measures may introduce response bias and social desirability effects. Therefore, future research should incorporate objective performance indicators and multi-method assessment approaches to strengthen the validity of findings (Paulhus and Vazire, 2007).

A review of the literature indicates that there are relatively few studies examining the effects of PSI interventions on the tactical skills of football players. For example, Pepe et al. (2020) provided training focused on mental toughness and decision-making skills to football players competing in the U-19 category of professional football teams and evaluated their competitive performance. The results of the study indicated that the training implemented with the experimental group positively influenced the players’ competitive performance. Similarly, Ballıkaya and Saraç (2024) implemented a 12-week PSI program with high school–aged volleyball players and reported significant improvements in the athletes’ coping skills and performance strategies.

Güneş and Yılmaz (2019) reported significant differences in favor of the experimental group in the decision-making, support, participation, and game performance components following basketball training conducted using a tactical games approach in physical education classes. Similarly, Figueira et al. (2022) implemented a video modeling intervention (e.g., observing match or scenario-based videos) with young basketball players and compared pre-test and post-test measurements during a four-week training period. The results indicated that the experimental group demonstrated significant improvements in technical-tactical performance indicators, such as passing accuracy, offensive effectiveness, and reduction in point loss, compared with the control group. Mamassis and Doganis (2004), in their study examining the effects of a Psychological Skills Intervention (PSI) program on tennis performance, reported that tennis players who participated in mental training were able to overcome performance-related problems, which consequently led to an overall improvement in their performance levels. Similarly, Weinberg (2001), in a review of research on the effects of various mental training strategies on athletic performance, concluded that mental training interventions are generally effective in enhancing performance outcomes. Furthermore, Bar-Eli et al. (2002), in a study investigating the effects of biofeedback-based mental training on the performance of young swimmers, divided participants into experimental and control groups and reported that swimmers in the experimental group demonstrated greater performance improvements compared to those in the control group.

In their study examining the effects of mental training on the acquisition of gymnastics skills, Alshadida and Al-Khasawneh (2024) reported that integrating mental training into athletes’ training programs is highly effective in facilitating skill acquisition and accelerating learning. Accordingly, the authors emphasized the importance of supporting athletes’ training programs with structured mental training interventions. Overall, these findings demonstrate that the psychological skills intervention program is effective in improving both offensive and defensive tactical skills, and that the improvement in tactical performance was more pronounced in the experimental group than in the control group. Furthermore, a review of the literature indicates that studies examining the relationship between decision-making, psychological stress, and tactical behavior identify football players’ psychological stress levels and decision-making abilities as important determinants of tactical effectiveness (García et al., 2024; Arısoy, 2019). Similarly, García et al. (2024) reported that PSI programs led to improvements in football players’ understanding of game principles and in their tactical behavior. Such holistic interventions enhance football players’ in-game performance efficiency by simultaneously addressing both tactical behavior and psychological components.

The role of Psychological Skills Intervention (PSI) programs in improving tactical skills can be explained by their capacity to integrate psychological skills with cognitive–tactical behaviors during football players’ in-game adaptation and decision-making processes. This integration enables players to demonstrate more effective, conscious, and adaptable tactical behaviors on the field (García et al., 2024; Roca et al., 2012; Gonçalves et al., 2017).

## 5 Conclusion

The findings of the present study support previous research indicating that structured psychological skills training can enhance athletes’ mental toughness and cognitive performance (Gucciardi et al., 2017). Improvements in decision-making and tactical execution may be explained by enhanced attentional control and emotional regulation, which are essential for adapting to dynamic game situations in football.

Although the findings generally support the proposed hypotheses, it is important to note that the causal mechanisms implied in the conceptual model were not directly tested mediation or structural equation modeling. Therefore, interpretations regarding indirect effects should be considered theoretical rather than empirically confirmed.

In conclusion, the eight-week Psychological Skills Intervention (PSI) program had a positive impact on the mental toughness and tactical skills of U-19 football players, particularly in the experimental group. Athletes in the experimental group demonstrated significant improvements in both mental toughness and offensive and defensive tactical skills. No statistically significant differences were observed between the control and experimental groups during the pre-test phase, either on the overall scales or across the sub-dimensions, indicating baseline equivalence between the groups. While significant increases were observed in the control and constancy sub-dimensions of mental toughness in the experimental group, self-confidence scores decreased in both groups during the post-test period. Furthermore, the group × time interaction effects provided evidence supporting the effectiveness of the PSI program. When effective decision-making skills were examined, both the control and experimental groups showed increases over time in the internal and external decision-making sub-dimensions; however, the improvement observed in the experimental group was more consistent and pronounced. In terms of tactical skills, the experimental group demonstrated significant increases over time in both offensive and defensive dimensions, and the group × time interaction effects indicated the effectiveness of the PSI program. Specifically, in offensive tactical skills, significant and consistent improvements were observed in the experimental group in positioning, decision-making, and awareness of ball actions. Similarly, in defensive tactical skills, improvements were found in recognizing other players and responding effectively to changing situations. These findings suggest that PSI programs can be considered an effective method for both enhancing the mental toughness and improving the tactical skills of young football players. It is therefore believed that integrating PSI programs as a systematic and integral component of training programs for footballers competing in development leagues can significantly support players’ individual performance and career development as they progress through the competitive pathway. Furthermore, such programs may play a critical role in developing elite-level footballers capable of contributing to the sustainable success of national football.

## 6 Recommendations

### 6.1 Theoretical implications

The findings of this study demonstrate that Psychological Skills Intervention (PSI) programs influence football performance not only at the psychological level but also within a multidimensional framework encompassing cognitive and tactical processes. In this context, it is suggested that approaches in the sport psychology literature that primarily associate PSI programs with direct performance outcomes should be reconsidered within a broader perspective. The results of the present study support the notion that PSI programs strengthen mental toughness, that this development optimizes decision-making processes, and ultimately contributes to more effective tactical performance on the field. Therefore, future theoretical models should address the impact of Psychological Skills Intervention (PSI) programs on performance through mediated mechanisms, positioning mental toughness and decision-making processes as fundamental psychological and cognitive antecedents of tactical performance. Furthermore, in sports such as football, which involve time pressure and uncertainty, performance cannot be explained solely by physical and technical competencies. Instead, perceptual–cognitive expertise and psychological resilience should be more strongly integrated into theoretical models of performance. Additionally, future research should aim to include larger and more diverse samples, apply longitudinal designs, and integrate objective performance metrics to further validate and extend the current findings.

### 6.2 Practical implications: a structured model for coaches and athletes

The findings of this study demonstrate that Psychological Skills Intervention (PSI) programs are effective in developing football players’ mental toughness, decision-making abilities, and tactical skills. Accordingly, a structured implementation model that encourages the active involvement of both coaches and athletes is proposed to effectively translate PSI programs into practical field applications.

### 6.3 Practical recommendations for coaches

The findings of the present study provide important practical implications for coaches, sport psychologists, and football training programs. Integrating Psychological Skills Intervention (PSI) programs into regular training routines may enhance athletes’ mental toughness, decision-making abilities, and tactical performance. These findings suggest that psychological training should be considered an essential component of athlete development alongside physical and technical training.

Coaches play a central role in the successful implementation of PSI programs. Therefore, psychological skills applications should not be considered solely as independent practices conducted under the responsibility of sport psychologists, but also as an integral component of the overall training process. In this regard, it is recommended that coaches allocate specific time slots within weekly training schedules for PSI-related activities and integrate these practices with technical and tactical training sessions. Particularly in training activities such as small-sided games, transition drills, and exercises that require decision-making under pressure, the simultaneous use of techniques such as breathing control, attentional focus, and positive self-talk alongside game-related tasks can strengthen the transfer of psychological skills into actual performance contexts. Additionally, organizing brief feedback sessions after training and competitions can help athletes become more aware of their decision-making processes and mental responses, thereby enhancing the retention and consolidation of learning. Furthermore, it is recommended that coaches participate in in-service training programs to develop fundamental knowledge and awareness regarding PSI programs. Such training can promote consistency in terminology and application when transferring psychological skills to athletes. This approach may also strengthen the sense of trust within the coach–athlete relationship and support the development of athletes’ mental toughness.

### 6.4 Practical recommendations for athletes

For practitioners, implementing structured PSI programs may contribute to improved performance consistency and cognitive efficiency under competitive pressure. For researchers, future studies should explore the underlying mechanisms of these effects using advanced statistical models such as mediation or structural equation modeling.

For athletes, the effectiveness of PSI programs depends on the conscious application of psychological skills not only during structured training sessions but also throughout daily training and competition routines. In this regard, it is recommended that athletes actively apply techniques such as breathing exercises, imagery (visualization), and positive self-talk not only during stressful situations but also during pre-training preparation, in-game focus, and recovery following mistakes. Particularly in decision-making processes, athletes’ ability to mentally visualize in-game scenarios and evaluate potential options can enhance their tactical awareness. In addition, maintaining individual performance diaries in which athletes reflect on their mental states, decision-making processes, and tactical applications following training sessions and competitions can contribute to the development of self-awareness and self-regulation skills. Furthermore, fostering positive and supportive patterns of internal and external communication within the team can support the development of mental toughness not only at the individual level but also at the team level. It is therefore important for athletes to perceive PSI programs not as short-term interventions, but as integral components of their long-term career development.

### 6.5 Practical recommendations for coach–athlete interaction

To enhance the effectiveness of PSI programs, it is recommended that shared goals and a common communication framework be established between coaches and athletes. During training planning, psychological objectives should be clearly defined alongside technical and tactical goals, and performance evaluations should consider not only physical and technical outcomes but also indicators related to decision-making quality and mental toughness. Adopting such a holistic approach can facilitate the effective translation of PSI programs into on-field performance, enabling players to make better decisions under pressure and demonstrate their tactical skills more consistently.

### 6.6 Recommendations for future research

While the findings of this study provide important contributions, several methodological and theoretical extensions are recommended for future research. First, examining the effects of PSI programs through long-term follow-up measurements would be valuable for assessing the sustainability of the improvements achieved. Furthermore, comparative studies across different age groups (e.g., U-15, U-17, and senior teams) and competition levels (amateur vs. professional) could provide a clearer understanding of the developmental impact of PSI programs. Future research is recommended to incorporate objective performance indicators such as game analysis data, GPS-based performance metrics, and match statistics in addition to self-report scales. Furthermore, examining mental toughness and decision-making variables through sequential or serial mediation models may provide a deeper understanding of the mechanisms through which PSI programs influence performance outcomes. Finally, multicenter and international studies investigating the role of cultural context in the effectiveness of PSI programs are expected to make significant contributions to the literature.

## 7 Limitations

One of the primary limitations of this study is the relatively small sample size, which consisted of participants from only two football teams. This limitation may restrict the generalizability of the findings to broader populations of football players. Although randomized group assignment strengthens internal validity, external validity remains limited. Future studies should include larger and more diverse samples from different competitive levels and cultural contexts to enhance generalizability (Bornstein et al., 2013).

The self-report scales employed in this study are subject to several methodological limitations. First, due to social desirability bias, participants may refrain from disclosing their genuine thoughts, feelings, and behaviors, instead providing responses they perceive as more socially acceptable (Fisher, 1993). This tendency is particularly problematic when addressing sensitive topics, as it can reduce the accuracy of the data (Krumpal, 2013). Moreover, because self-report techniques rely on individuals’ subjective perceptions, cognitive constraints (e.g., recall errors, selective memory, exaggeration), and motivational factors (e.g., the tendency to present oneself in an overly positive or negative manner) can introduce systematic biases (Chan, 2009). In addition, participants’ personality traits, current mood, attentional focus, and the way questions are formulated may all influence the consistency of responses (Paulhus and Vazire, 2007; Podsakoff et al., 2003). Collectively, these factors can restrict the objectivity and internal validity of the findings, thereby limiting the generalizability of the results (Tourangeau and Yan, 2007).

This study was conducted only with football players who met the following criteria: (i) being over 18 but under 20 years of age; (ii) actively competing in the U-19 category; (iii) having at least 3 years of licensed football experience; and (iv) completing the voluntary consent form.

The research was conducted on a sample group, which has the limitation of not representing the entire population. Therefore, similar studies with different sample groups should be conducted. Since this study was conducted only on football players competing U-19 of professional football teams, the results may not be generalizable to other age categories.

The personal opinions and experiences of the study participants, as well as their tendency to present themselves in a more positive light, may impact the generalizability and objectivity of the findings. Therefore, a similar study should be conducted longitudinally. Data were collected entirely via self-report measures (online surveys), which introduces potential limitations such as social desirability bias and standard method variance. Participants’ personal opinions, self-perceptions, and their tendency to present themselves in a more positive light may have influenced the objectivity of the responses. To address this, future studies are recommended to employ multi-source data collection methods (e.g., observations, football coaches or football managers).

The PSI program is limited by its application over only 8 weeks, the lack of knowledge regarding its long-term effects, the failure to control external factors such as training intensity, nutrition, sleep, and psychological state, and the fact that measurements focus solely on mental toughness, self-confidence, and tactical skills, thereby neglecting to evaluate other factors such as physical performance, motivation, perception, and psychological stress.

In addition, as the data were collected through self-reported questionnaires, there is a potential limitation related to the tendency of participants to respond in a socially desirable manner. In light of these limitations, future studies should employ longitudinal designs, collect data from multiple sources, and replicate the findings with different national and cultural samples to validate and extend these results.

## Data Availability

The original contributions presented in the study are included in the article/supplementary material, further inquiries can be directed to the corresponding authors.

## Footnotes

1
www.random.org
